# Expression profiling and intracellular localization studies of the novel Proline-, Histidine-, and Glycine-rich protein 1 suggest an essential role in gastro-intestinal epithelium and a potential clinical application in colorectal cancer diagnostics

**DOI:** 10.1186/s12876-018-0752-8

**Published:** 2018-02-07

**Authors:** Satu Oltedal, Ivar Skaland, Jodi Maple-Grødem, Kjersti Tjensvoll, Emiel A. M. Janssen, Bjørnar Gilje, Rune Smaaland, Reino Heikkilä, Oddmund Nordgård

**Affiliations:** 1 Department of Hematology and Oncology, Stavanger University Hospital, 4068 Stavanger, Norway; 2 Department of Pathology, Stavanger University Hospital, 4068 Stavanger, Norway; 3 The Norwegian Centre for Movement Disorders, Stavanger University Hospital, 4068 Stavanger, Norway; 4 Centre for Organelle Research, University of Stavanger, 4036 Stavanger, Norway; 5 Oslo University Hospital, 0424 Oslo, Norway

**Keywords:** Colon, Mucosa, Glycoprotein, Transport, Metastasis, Cancer

## Abstract

**Background:**

The primary function of the intestines is the absorption of water and nutrients. Although our knowledge about these processes on the cellular level is extensive, a number of important intracellular elements remain unknown. Here, we characterize the novel proline-, histidine-, glycine-rich 1 (PHGR1) mRNA and protein on the molecular level and propose a functional role of the PHGR1 protein in the intestinal and gastric epithelium.

**Methods:**

PHGR1 mRNA and protein expression in human tissues and cell lines were characterized by quantitative RT-PCR, in situ hybridization, Northern blotting, Western blotting, and immunohistochemistry. Glycosylation was assessed by a chemical deglycosylation assay, whereas intracellular localization was studied by immunofluorescent staining of cell line cells. PHGR1 mRNA levels in HT29 cells was reduced by RNA interference and the resulting global changes in gene expression assessed by microarray hybridization.

**Results:**

PHGR1 mRNA and protein were found to be expressed specifically in epithelial cells of intestinal mucosa, with the highest expression in the most mature and differentiated cells. PHGR1 protein was found to be glycosylated and to localize to both the cytoplasm and nucleus. Transcript profiling and gene ontology analysis of HT29 cells subjected to PHGR1 knockdown suggested a functional relationship with transport and metabolic processes. Examination of PHGR1 mRNA and protein levels in lymph nodes with known colorectal cancer metastases indicated that they may serve as biomarkers for detection of such metastases.

**Conclusions:**

Functional analyses of the novel PHGR1 mRNA and protein suggest an essential role in gastrointestinal epithelium and a clinical application in detection of colorectal cancer lymph node metastases.

**Electronic supplementary material:**

The online version of this article (10.1186/s12876-018-0752-8) contains supplementary material, which is available to authorized users.

## Background

The intestinal mucosa forms an important barrier against nutrients and microorganisms within the intestines. Other primary functions of the mucosa are absorption, secretion, and maintaining symbiosis with the intestinal microbiota [1].

The intestinal mucosa is covered by a single cell layer consisting primarily of four cell types [1, 2]. The most abundant cells are the absorptive *enterocytes*; *goblet* cells and *enteroendocrine* cells secrete mucus and various hormones; and *Paneth* cells secrete bactericidal proteins, such as lysozyme and defensins. The mucosal cell layer forms crypts, and in the small intestine protrusions called villi are formed, maximizing the surface area. Mature enterocytes, goblet cells, and enteroendocrine cells cover the villi of the small intestine and the luminal surface of the large intestine. Paneth cells and immature enterocytes, goblet, and enteroendocrine cells reside in the basal parts of the crypts, which is a niche of proliferation and cell differentiation. The bottom of each crypt also harbors stem cells that give rise to all of the cell types described above [2].

The enterocytes are highly polarized columnar cells with an apical surface facing the lumen and a basolateral surface connecting the mucosa to the other cell layers of the intestinal wall [3]. The apical surface is covered by numerous membrane protrusions, called the brush-border, further maximizing the surface area and absorptive capacity of the cell. The brush-border is covered by layers of mucus and glycocalyx, which provides protection and contains digestive enzymes [1]. Substantial membrane and cytoplasmic asymmetry is required to maintain the brush-border structure and facilitate the transport of nutrients and water from the intestinal lumen to the basolateral surface. Membrane asymmetry is generated and maintained primarily by vesicular transport, including both specific transport of newly synthesized proteins from the trans-Golgi network and recycling of existing membrane proteins via recycling endosomes [3, 4].

Vesicular transport is also responsible for the direct transport of some nutrients across the mucosal cell layer in a process called transcytosis [4]. However, the gross absorption of nutrients, electrolytes, and water from the lumen is performed by specific membrane-spanning transporter proteins [5]. Specific transporters also secrete water and electrolytes, maintaining an intricate chemical homeostasis both inside the cells and in the neighboring compartments.

Enterocytes also play a role in the immunological defense system of the gut [1], as they contribute to the activation of the immune system against disease-causing microorganisms and to the suppression of immune reactions against the beneficial inhabitants of the gut. These activities include uptake and presentation of antigens, interaction with immune cells and secretion of immunoglobulin A and antimicrobial peptides [1].

Even if many aspects of intestinal biology are well-described, important details are still lacking regarding the level of molecular mechanisms, signaling, and integration of processes. Through bioinformatic searches for colorectal cancer biomarkers, we identified the novel gastrointestinal-specific proline-, histidine-, glycine-rich protein 1 (PHGR1). Here, we describe the first molecular characterization of PHGR1 and suggest a clinical utility in colorectal cancer diagnostics.

## Methods

### PHGR1 5′ end cDNA cloning and sequencing

The complete 5′ end of *PHGR1* cDNA was cloned by RNA ligase-mediated rapid amplification of 5’ cDNA ends (RLM-RACE), using the GeneRacer kit (Invitrogen) according to the kit protocol. The 5′ end of *PHGR1* cDNA was then PCR amplified, using one adapter-specific primer and a *PHGR1*-specific primer with the following sequence: 5′- TTC TCT CAG GCT TGC CAT CTT GT -3′. The PCR products were cloned using the Zero Blunt TOPO PCR Cloning Kit for Sequencing (Invitrogen). DNA sequencing was performed using the BigDye Terminator v3.1 Cycle sequencing Kit (Applied Biosystems) and capillary electrophoresis at the sequencing facility of the SARS center in Bergen. We sequenced four clones, from two different individuals, and they were identical. Our *PHGR1* mRNA sequence (5′ end) was uploaded to Genbank (Genbank: KT007223).

### Cell culture

Caco-2, LS174T, HT29, and HCT116 cells were cultured according to the recommendations of the provider (ECACC).

For the differentiation experiments, non-confluent Caco-2 cells were plated in each well of 12-well culture plates, corresponding to a density 2.9 x 10^4^ cells/cm^2^ and approximately 50% confluence. The culture medium was exchanged every second day until the last cultures were harvested after 14 days. The cells reached confluence at day 2. After 0, 1, 2, 4, 6, 8, 10, and 14 days of growth, three cultures were washed with phosphate-buffered saline (PBS) (37 °C) and lysed in 350 μl of RLT buffer (from the Allprep DNA/RNA/Protein Mini kit, Qiagen).

For the intracellular localization studies Caco-2 cells were seeded at a density of 2.9 x 10^4^ cells/cm^2^ in 12-well transparent polyethylene terephthalate (PET) hanging cell culture filter inserts (Millipore) with 1.0 μm pores. The cells reached confluence at day 2 and the medium was exchanged every second day until harvesting. Parallel cultures were stained according to the protocol in the “Immunofluorescent staining” section or lysed in RLT buffer for RNA isolation.

### Northern blotting

RNA (5 μg) from the LS174T cell line, a colorectal adenocarcinoma sample and a normal colonic mucosa sample were subjected to agarose gel (1.2%) electrophoresis using MOPS electrophoresis buffer and 2% formaldehyde in the gel, according to the “DIG Application Manual for Filter Hybridization” (Roche). A digoxigenin (DIG)-labeled RNA size standard was analyzed in parallel. RNA was blotted onto a positively charged nylon membrane by capillary transfer and hybridized to 2.5 pmol of four PHGR1-specific DIG-labeled DNA hybridization probes, according to the same DIG application manual. The sequences of the hybridization probes were: 5′-GGA GTA AGA GCA GGG AAT ACC TGA GAG TGC AGA GCA GGG GCA GGA AGT C-3′, 5’-GCC CGC AGT GAC CTG GAG GAT GGC CAT GCC CCC CAC AGT G-3′, 5’-GGC CCT GGA CCA TGG TGG GGG GGT GGC CCG CAG G-3′ and 5’-GGC CTC ATT CCA GGT CAG CTG GGC AAT TCT CTC AGG CTT GCC-3′. The hybridization signals were detected by an alkaline phosphatase-conjugated DIG antibody according to the application manual.

### In situ hybridization

In situ hybridization was performed essentially as described in the DIG Application Manual for Nonradioactive In Situ Hybridization (3rd edition, Roche). DIG-labeled cRNA probes were prepared using a DIG-RNA-labeling kit (Roche). The antisense and probe mixture consisted of the same four probes as described in the Northern blotting section, 20 ng of each probe per slide. The sense probe mixture (negative control) consisted of four probes, 20 ng of each probe per slide. Unbound RNA probe was not digested, but removed by washing at 37 °C five times for 15 min each in decreasing concentrations of SSC buffer. The hybridization signals were detected by an alkaline phosphatase-conjugated DIG antibody according to the DIG Application Manual.

### RNA and protein purification

RNA and protein were isolated from cell cultures and tissue samples using the Allprep DNA/RNA/Protein Mini kit according to the manufacturer’s protocol (Qiagen). Protein pellets from mucosa tissue samples required 8 M urea/2% SDS and incubation at 70 °C for 2–3 h to dissolve.

### DNAse-treatment and reverse transcription

RNA was treated with DNAse by mixing 500 ng total RNA from each sample with 1 unit RQ1 RNAse-free DNAse (Promega) in a total volume of 20 μl First Strand Synthesis buffer (Invitrogen) containing 10 units RNAseOUT RNAse inhibitor (Invitrogen). The reaction mixture was incubated at 37 °C for 30 min and the DNAse inactivated by adding 1 μl RQ1 stop solution and incubating for 10 min at 65 °C. Complementary DNA was synthesized by M-MLV reverse transcriptase in a total volume of 40 μl according to the manufacturer’s protocol (Life Technologies).

### Quantitative PCR

PCR amplification of *PHGR1, HPRT1,* and *BCR* cDNA was performed with the qPCR SYBR Green Core kit (Eurogentec) according to the manufacturer’s recommendations. Thermocycling and real-time fluorescence measurements were performed in an Mx3000P real-time PCR instrument (Stratagene). The DNA sequences of the PCR primers were: PHGR1-F: 5’-CCCTGCTCTGCACTCTCAG-3′, PHGR1-R: 5’-CGCAGTGACCTGGAGGAT-3′, HPRT1-F: 5’-TTCCTTGGTCAGGCAGTA-3′, HPRT1-R: 5′- TATCCAACACTTCGTGGG-3′, BCR-F: 5’-GCTCTATGGGTTTCTGAATG-3′, BCR-R: 5’-AAATACCCAAAGGAATCCAC-3′.

Relative levels of *PHGR1* mRNA were determined by normalization against a reference transcript *(HPRT1* or *BCR*) and a calibrator sample according to the 2^ΔΔCq^ method [6]. All analyses were performed in duplicates or triplicates.

*PHGR1* mRNA expression profiling was done by measuring the relative *PHGR1* mRNA levels in 2 μl of cDNA from Multiple Tissue cDNA Panels I and II (BD Bioscences), cDNA from normal colon mucosa biopsies and lymph nodes from healthy controls described below. The means from three independent experiments were presented.

*ALPL, MAPK3, MAPK13, SLC2A1, SLC44A2, S100A3, SPRR1A, DDIT4, RASD1*, and *SLC4A* mRNA levels were measured relative to *HPRT1* mRNA using TaqMan assays (Thermo Fischer), according to the manufacturer’s protocol.

### Antibody synthesis

A polyclonal rabbit antibody against a synthetic subpeptide of the PHGR1 protein was raised in two rabbits through a custom antibody production service (Eurogentec). The peptide sequence of the synthetic subpeptide was N-CGPPPGHGPGHPPPGP-C, which is part of the repeated region of PHGR1. The antibody was affinity purified from antiserum after the final bleed.

### Western blotting

Proteins were separated according to size by SDS-PAGE using 15% polyacrylamide gels and a Tris-glycine buffer system in a Mini-Protean electrophoresis unit (Bio-Rad). Separated proteins were blotted onto PVDF membranes (Bio-Rad Immunoblot) by semi-dry electro-blotting for 20 min at 15 V using a Transblot SD unit (Bio-Rad). The anode filter paper (Extra thick blot paper, Bio-Rad) and membrane were soaked in 25 mM Tris base/20% methanol, whereas the cathode filter paper was soaked in 25 mM Tris base/20 mM 6-Aminocaproic acid/20% methanol. Proteins were fixed to the membranes by incubation in methanol for 2 min and reversibly stained with Ponceau stain to validate blotting quality. The membranes were blocked by incubation for 30 min with shaking in TBS-T (10 mM Tris-HCl pH 8.0, 0.15 M NaCl, 0.05% Tween-20) buffer added 5% skimmed milk powder, then shaken for two hours at room temperature with primary antibodies (rabbit anti-PHGR1 1:2000 and rabbit anti-GAPDH (Abcam) 1:1000) in TBS-T/milk buffer. Subsequently, the membranes were washed 3 × 20 min in TBS-T/milk buffer and incubated for one hour of shaking with an alkaline phosphatase-conjugated goat antibody against rabbit immunoglobulin G (Sigma-Aldrich), diluted 1:10,000 (PHGR1) or 1:5000 (GAPDH) with TBS-T/milk. Finally, the membranes were rinsed 6 times with TBS-T, washed for 20 minutes with TBS-T, incubated for one minute in 0,1 M Tris-HCl pH 9.5/0,1 M NaCl and overlaid with BCIP/NBT purple liquid substrate system for membranes (Sigma Aldrich) in darkness. Color reactions were stopped after sufficient signal development (5–15 min) by rinsing with distilled water.

### Chemical deglycosylation

The protein fraction was extracted from 5 to 10 million LS174T cells by the Allprep DNA/RNA/protein Mini kit according to the manufacturer’s protocol (Qiagen) and dissolved in 5% SDS. The SDS was subsequently removed by purification on PD Spintrap G-25 gel filtration spin columns (GE Healthcare) using milli-Q water as the equilibration buffer in accordance with the manufacturer’s protocol. The water was removed by vacuum centrifugation. Protein extracts were chemically deglycosylated using the GlycoProfile IV chemical deglycosylation kit (Sigma-Aldrich) according to the manufacturer’s protocol. In short, 75 μl pre-cooled (2-8 °C) Trifluoromethanesulfonic (TFMS) acid was added to pre-cooled dried protein extracts. The reaction tubes were immediately sealed and proteins dissolved by gentle shaking for 2–5 min. The samples were incubated on ice for 25 min and added pre-cooled (methanol-dry ice bath) pyridine solution dropwise until the pH reached approximately 6 (bromophenol blue turned blue). The reaction vessel was cooled on methanol-dry ice water bath during the first phase of pyridine addition to avoid freezing. Finally, deglycosylated proteins were dialyzed using the PlusOne Mini Dialysis kit (GE Healthcare), concentrated by vacuum centrifugation, and analyzed by Western blotting as described above. The volume of deglycosylated protein extract loaded on the acrylamide gel for SDS-PAGE corresponded to the volume of undeglycosylated protein extract loaded, corrected for dilutions and up-concentration during deglycosylation. The experiment was repeated four times, two times according to kit protocol A and two times according to protocol B (using anisole scavenger), producing the same results in all replicates.

### Immunofluorescent staining

Caco-2 cells grown on PET hanging cell culture filter inserts (Millipore) were washed in PBS, fixed in 4% paraformaldehyde, permeabilized, blocked and incubated for 1 h at room temperature in a 1:500 dilution of our rabbit polyclonal PHGR1 antibody in blocking solution or an isotype-specific control antibody for polyclonal rabbit antibodies (Life Technologies 08–6199). The cells were washed and incubated for 30 min in a 1:2000 dilution of the secondary antibody (goat polyclonal FITC-conjugated antibody against rabbit IgG, Sigma-Aldrich). The cells were subsequently washed and incubated for 30 min with a 1:500 dilution of a mouse monoclonal Alexa Fluor 594-conjugated antibody against occludin (Life Technologies). The cells were then washed and incubated for 10 min in a 0.2 μg/ml dilution of 4′,6-diamidino-2-phenylindole dihydrochloride (DAPI, Sigma-Aldrich). The filters were removed from the inserts using a scalpel and mounted on glass slides with fluorescent mounting medium (Dako). Pictures were obtained at room temperature with an Axioplan 2 fluorescence microscope (Carl Zeiss) and a JAI M4+ HiRes Digital CCD camera, using fixed exposure times (1 s.) for the green and red channels and automatic exposure times for the blue channel. The objectives were Carl Zeiss 10× Plan-APOCHROMAT /0.45 NA and 100× Plan-NEOFLUAR/1.3 NA oil. The software ISIS version 5.2.23 was utilized for capture and image processing. Lower signal thresholds were the same for all images obtained with the same magnification. The images shown in Fig. 4 and Additional file 3 are representative of the majority of the investigated Caco-2 cells.

Images were also taken using an inverted Nikon A1R confocal laser scanning microscope with a 60X Plan-Apo/1.20 NA oil objective. Excitations used laser lines at 408 nm, 488 nm, or 561 nm, and images were recorded at 450/50 nm, 525/50 nm, or 595/50 nm, respectively. Stacks of images were acquired with a 0.2 μm confocal slice. NIS-Elements imaging software 4.0 (Nikon, Japan) was used for image capture, and the brightness of the resulting pictures was increased by adjusting the signal threshold.

### Immunohistochemistry

Paraffin-embedded tissue sections were mounted onto Superfrost Plus slides (Menzel, Braunschweig, Germany) and dried overnight at 37 °C followed by 1 h at 60 °C. The sections were deparaffinized in xylene and rehydrated in decreasing concentrations of alcohol. Antigen was retrieved using a highly stabilized retrieval system (ImmunoPrep, Instrumec, Oslo, Norway) at high temperature and pressure. The sections were incubated for 30 min with a 1:20,000 dilution of our rabbit polyclonal PHGR1 antibody in Dako antibody diluent. The EnVisionTMFlex detection system (Dako) was used for visualization. After washing the sections, they were incubated for 5 min with peroxidase-blocking reagent, 20 min with the EnVision™ FLEX/HRP Detection Reagent, 10 min with EnVision™ FLEX DAB+ Chromogen/EnVision™ FLEX Substrate Buffer mix and 5 min with EnVision™ FLEX Hematoxylin. The slides were dehydrated and mounted. All immunohistochemical stainings were performed using a Dako Autostainer Link 48 instrument and EnVision™ FLEX Wash Buffer. Micrographs were obtained with an Olympus IX81 microscope and a MMI CellCamera 1.4 camera, using Olympus 20× LUCPlanFLN/0.45 NA and 100× UPlanSApo/1.4 NA oil objectives. The MMI CellTools-4-4 software was used.

### PHGR1 knockdown in HT29 cells

Proliferating HT29 cells were plated in 12-well culture plates at a density of 30,000 cells per well and cultured overnight without antibiotics. These cells were then transfected separately with three different small interfering RNAs (siRNAs) in quadruplicates using Dharmafect 4 transfection reagent (Dharmacon/GE healthcare), according to the manufacturer’s protocol. Two siRNAs (A and B) were targeted against PHGR1, whereas the third was a negative control (C). The siRNA sequences were siPHGR1-A: 5’-GAUGGCAAGCCUGAGAGAA(dTdT)-3′ (MWG Biotech siMAX), siPHGR1-B: 5’-AGAUGGACCCAGGUCCGAA(dTdT)-3′, and a negative control siRNA: AGGUAGUGUAAUCGCCUUG(dTdT)-3′ (scramble). Untransfected control wells were also included. Subsequently, the cells were cultured for 48 h and lysed in RLT buffer containing β-mercaptoethanol. RNA was isolated with the RNeasy Mini kit according to the kit protocol, using the optional on-column DNAse digestion step. The RNA quality was verified on the Bioanalyzer 2100 (Agilent).

PHGR1 mRNA knockdown was confirmed by quantitative RT-PCR as described above. A separate, identical transfection experiment was performed to verify the reduced PHGR1 protein level upon siRNA transfection. The cells were then lysed and analyzed by Western blot according to the protocol described above.

### Global mRNA profiling and data analysis

One microgram of RNA from each transfected HT29 culture was amplified and labeled using the Illumina RNA Amplification kit and hybridized to Illumina Human-6 Expression BeadChips (48,000 different probes and 1.6x10^6^ beads). Fluorescence was measured in a BeadArray Reader (Illumina). Amplification, hybridization and fluorescence measurements were performed by the Norwegian Microarray Consortium (NMC) in Oslo.

Data extraction and initial quality control were performed using BeadStudio version 3.1.3.0 from Illumina (http://www.illumina.com) and Gene Expression module 3.2.6. Additional quality control and statistical and gene ontology analysis were performed using the R (version 3.0.2, http://www.r-project.org) and Bioconductor (version 2.22.0, http://www.bioconductor.org) software packages [7, 8]. Raw fluorescence values were quantile normalized, log-transformed and submitted to the Gene Expression Omnibus (GEO) database (GEO: GSE70053). Differential gene expression between samples was assessed with the limma package in Bioconductor. *P*-values were adjusted for multiple testing by the Benjamini and Hochberg method [9]. Transcripts with significantly different levels (adjusted *P* < 0.001) in both comparison groups (A vs. control and B vs. control) were considered to be significantly upregulated and downregulated.

Gene ontology analysis was performed using the Database for Annotation, Visualization and Integrated Discovery (DAVID) [10] web service version 6.7 and GOstat [11] version 2.28.0 for Bioconductor. Similar results were obtained with both methods, but only the DAVID results are shown in the article. The gene ontology annotations of significantly upregulated or downregulated genes were compared with the annotations for all genes analyzed in the mRNA profiling, in order to identify over- or underrepresented gene ontology categories. *P*-values were adjusted for multiple testing by the Benjamini-Hochberg method. Under-represented categories were only assessed by the GOstats package (not shown), but were complementary to the over-represented categories.

### Other bioinformatics

Expressed sequence tag (EST) databases at the Cancer Gene Anatomy Project (CGAP; US National Cancer Institute) were searched for potential colorectal cancer biomarkers using the cDNA digital gene expression displayer (DGED) tool in June 2004. Libraries from normal colon and colon cancers (including cell lines) were compared to all other available libraries.

Multiple sequence alignments were analyzed using MUSCLE 3.51 software. The alignment was colored by JalView version 2.7. Homology searches were performed with the blastn, blastp, tblastn, Phi-blast, PSI-blast, Delta-blast and HMMER3 software programs in the Genbank nr and Swissprot databases. The subcellular localization of PHGR1 was predicted by PSORT II. No clear predictions were possible from the primary sequence of PHGR1 (data not shown).

### Patients and control samples

Colon and lung tumor biopsies, normal mucosa, and lymph node biopsies were obtained from patients and controls recruited into two prospective clinical studies previously approved by the Norwegian Regional Ethical Committee West (approval 197.04) and the Norwegian Regional Ethical Commmittee South (approval S-05307) [12–14]. Written informed consents from the donors were obtained for the use of their tissue samples in research. The colon cancer study included operable colon cancer patients, and the lung cancer study included operable non-small cell lung cancer patients. The samples analyzed in Fig. 6a and b were selected from the existing biobanks by randomization. Clinicopathological data for the 209 colon cancer patients analyzed in Fig. 6b and c are shown in Additional file 8. RNA from other human tissues was purchased from BD Bioscences and Asterand Bioscience.

## Results

### In Silico identification and characterization of PHGR1

EST libraries at the Cancer Gene Anatomy project (CGAP) were searched for potential colorectal cancer biomarkers using the cDNA digital gene expression displayer tool, by comparing libraries for normal colon and colon cancers to all other available libraries. Among the mRNAs apparently only expressed in normal and neoplastic colon tissues were several well-known colonic differentiation markers and a novel mRNA later denoted as proline-, histidine-, glycine-rich protein 1 (PHGR1).

**Fig. 1:**
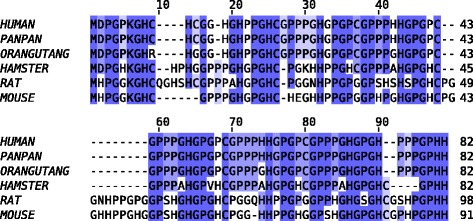
Multiple sequence alignment of PHGR1 orthologs. Predicted PHGR1 polypeptides from *Homo sapiens* (human), *Pan paniscus* (PanPan), *Pongo abelii* (orangutan), *Cricetulus griseus* (hamster), *Rattus norvegicus* (rat) and *Mus musculus* (mouse) were aligned by the MUSCLE multiple sequence alignment software. Identical residues across all peptides are shown in dark blue and conserved residues (positive BLOSUM62 score) in light blue

The gene and mRNA sequences of *PHGR1* from several species were available in Genbank and equivalent sequence databases. We verified experimentally that the human *PHGR1* mRNA sequence in Genbank had a complete 5′ end (Genbank: KT007223). The human RefSeq *PHGR1* mRNA has 434 bases (without the polyA tail) and consists of four exons (Genbank: NM_001145643). The human *PHGR1* gene is located on chromosome 15 band q15.1. Two alternative open reading frames (ORFs) have been suggested in the publicly available sequence databases, encoding either an 82 amino acid (aa) or a 93 aa polypeptide (human). Therefore, we performed multiple sequence alignments for both groups of predicted polypeptides and found the 82-aa ORF to be best conserved and probably also the one that is translated. The predicted PHGR1 peptide sequence has extraordinarily high proline (29%), histidine (18%) and glycine (31%) content. Fig. 1 shows a multiple sequence alignment of the predicted PHGR1 protein orthologs, revealing that many of the glycine, proline, and histidine residues were well conserved. In addition, three cysteines and a lysine residue were present in all orthologs. Sequence analysis also revealed five repeats of approximately 11 aa in the N-terminal part of the peptide (Additional file 1). The predicted molecular weight of the PHGR1 polypeptide was 7.7 kDa.

Homology searches were performed with both the peptide sequence alone and the multiple sequence alignment (Fig. 1) as a query. When using compositional adjustment, only weak similarities were found, probably because very few amino acids in PHGR1 are not proline, histidine, or glycine. However, without compositional adjustment we found several proteins with significant similarity to the repeat region of PHGR1, mostly histidine and proline-rich proteins of various classes. The most significant hits with functional annotations were histidine-rich glycoproteins from animals, proline-rich cell wall glycoproteins from plants, and salivary proline-rich proteins and proline-rich proteoglycans from mammals. Several of these proteins had sequence repeats in their proline-rich regions and many of them were secretory and had signaling peptides. However, no conventional signaling peptide was present in PHGR1.

### *PHGR1* mRNA characterization

**Fig. 2:**
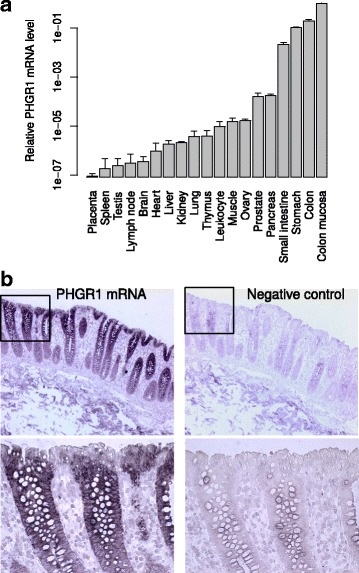
*PHGR1* mRNA characterization. **a** *PHGR1* mRNA levels in various tissues presented relative to a reference mRNA and the pooled colon mucosa samples. The error bars represent the standard deviation from three independent experiments. **b** In situ hybridization of *PHGR1* mRNA in the colon wall. The left panels show 10× and 40× (insert) microscopy images of a tissue section hybridized to *PHGR1-*specific probes. The right panels show corresponding images of an adjacent tissue section hybridized to a negative control probe. Nuclear counterstaining (blue) was achieved with hematoxylin

Northern blotting revealed a single *PHGR1* mRNA variant of approximately 490 bases in normal colonic mucosa, a colorectal tumor biopsy, and the colorectal cancer cell line LS174T (data not shown). This corresponded well to the RefSeq mRNA sequence of 434 bases without the polyA tail. The *PHGR1* mRNA expression profile in various normal tissues was determined by quantitative reverse transcription PCR (Fig. 2a). Very high levels of *PHGR1* mRNA were present in the intestines and stomach, but low levels were present in most other tissues. However, the prostate and pancreas had moderate levels of *PHGR1* mRNA.

To identify the colonic cell types with high levels of *PHGR1* mRNA, we performed *PHGR1* mRNA in situ hybridization in colon tissue biopsies (Fig. 2b). *PHGR1* mRNA was predominantly expressed in the upper parts of the crypts, where the more mature epithelial cells reside. Both the mucus-producing goblet cells and the absorptive enterocytes expressed high levels of *PHGR1* mRNA, whereas the mature epithelial cells facing the lumen of the colon had somewhat lower levels.

### PHGR1 protein characterization

**Fig. 3:**
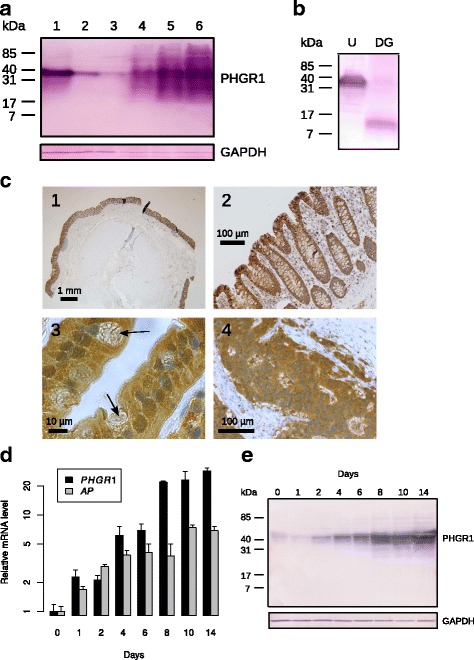
PHGR1 protein characterization. **a** Western blot of PHGR1 protein in the colorectal cancer cell lines LS174T (lane 1), HT29 (lane 2), and HCT116 (lane 3) and normal mucosa samples (lanes 4–6). GAPDH was included as a loading control. **b** Western blot of untreated (U) and chemically deglycosylated (DG) protein extracts from the LS174T cell line, stained with a polyclonal antibody against PHGR1. The volumes loaded corresponded to the same volume of original protein extract. **c** Immunohistochemical staining of PHGR1 in colon and colorectal cancer. 1) PHGR1-stained (brown) normal colon (2.5× microscope objective). 2) PHGR1-stained normal colon mucosa (20× objective). 3) PHGR1-stained normal mucosa (100× objective), upper part of a crypt. The arrows indicate goblet cells. 4) PHGR1-stained colorectal carcinoma (20× objective). Nuclear counterstaining was achieved with hematoxylin (blue). **d** Relative *PHGR1* and alkaline phosphatase (*AP*) mRNA levels during Caco-2 cell differentiation. mRNA levels were normalized both against the reference mRNA *HPRT1* and the day 0 sample. Data are represented as mean values from three biological replicates. The error bars represent standard deviation. **e** Western blot of Caco-2 cells cultured to confluence and beyond (0–14 days) using antibodies against PHGR1 (upper panel) and GAPDH (lower panel)

On Western blots using a polyclonal antibody against PHGR1 the LS174T, HT29, and HCT116 colorectal cancer cell lines and normal mucosa samples did not have a protein band around the expected molecular weight for PHGR1 (7.7 kDa). Instead, a strong band was observed near 40 kDa, in addition to a number of weaker bands with a larger size interval (Fig. 3a). PHGR1 mRNA levels were measured in the same samples, showing a remarkably good correlation with the protein levels (Additional file 2). PHGR1 protein levels were highest in the mucosa samples and the mucin-producing, goblet cell-like LS174T cell line [15] and almost absent in the low-differentiated and more aggressive HCT116 cell line. The HT29 cell line, which can differentiate into both enterocyte-like and mucin-producing lineages [16], had intermediate levels of PHGR1 protein.

Covalent modification of the PHGR1 peptide could explain its unexpectedly slow migration in SDS-PAGE. Further analyses showed that the PHGR1 peptides were resistant to trypsin digestion, but not proteinase K digestion. This finding was consistent with the presence of only one trypsin site at the very beginning (6th amino acid residue) of the predicted PHGR1 polypeptide (data not shown), which would probably not result in detectable changes in migration. This observation indicated that a covalently conjugated heterotypic protein complex was not a probable explanation for the apparently large size. The PHGR1 peptides seemed generally very stable, resisting incubation for several hours in a crude protein extract containing endogenous proteases without protease inhibitors (data not shown).

Variable protein glycosylation may be an alternative explanation for the numerous weaker bands observed in SDS-PAGE. Thus, we tried to remove the potential glycosylation of PHGR1 both enzymatically and chemically. However, the enzymatic deglycosylation did not result in any shift in electrophoretic mobility for the observed protein bands (data not shown). On the other hand, the chemical deglycosylation revealed a protein band at 10 kDa (Fig. 3b), supporting the hypothesis that PHGR1 is glycosylated.

Some proteins without a signaling peptide have been reported to be directed to secretion based on specific glycosylation [17]. However, we did not detect any PHGR1 in the growth medium of LS174T cells after 2 days of culture (data not shown).

### Immunohistochemical expression pattern of the PHGR1 protein

To investigate the pattern of PHGR1 protein expression, we performed immunohistochemical (IHC) staining of PHGR1 in normal colon, breast, liver, kidney, thyroid, pancreas, and placenta (Fig. 3c and Additional file 2). PHGR1 protein levels were high in colonic mucosa, but very low or undetectable in the other normal tissues tested. The PHGR1 protein levels were also highest in the upper parts of the colonic crypts, where the most mature epithelial cells reside, corresponding well with the expression pattern we observed for *PHGR1* mRNA.

Interestingly, IHC staining also revealed that the PHGR1 protein was present in both the cytoplasm and nucleus, with nuclear levels increasing more than cytoplasmic levels during differentiation. Accordingly, we analyzed several colorectal cancers and observed both cytoplasmic and nuclear localization in highly differentiated tumors. Tumors with a lower differentiation grade primarily had cytoplasmic localization of PHGR1 (Fig. 3c and data not shown). In general, both secretory goblet cells and absorptive enterocytes had high levels of PHGR1 protein. However, a few mature goblet cells with lower nuclear PHGR1 levels were also observed in normal mucosa (arrows in Fig. 3c).

### PHGR1 levels during enterocyte differentiation

When cultured beyond confluency, the Caco-2 human adenocarcinoma cell line expresses characteristics of enterocytic differentiation [18]. Therefore, to investigate *PHGR1* mRNA levels in a model of enterocytic differentiation, we measured *PHGR1* mRNA levels in differentiating Caco-2 cells by quantitative RT-PCR (Fig. 3d). We observed a strong increase in *PHGR1* mRNA levels during Caco-2 cell differentiation. The differentiation process was verified by measuring alkaline phosphatase mRNA levels in parallel. Moreover, Western blotting of protein lysates from the same Caco-2 cultures demonstrated a similar increase in the PHGR1 protein levels during differentiation (Fig. 3e).

### Intracellular localization of PHGR1 during enterocyte differentiation

**Fig. 4:**
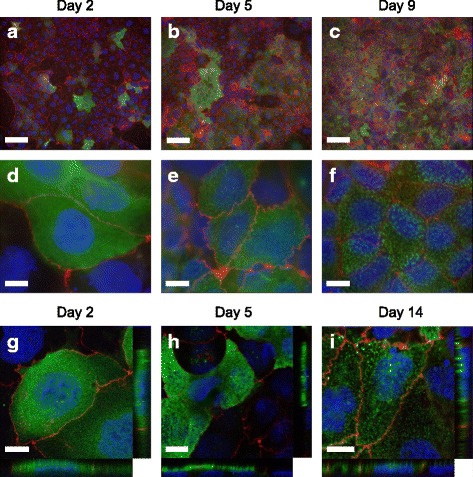
Immunofluorescent staining of PHGR1 protein in differentiating Caco-2 cells. Caco-2 cells grown on filters for 2 (**a, d, g**), 5 (**b, e, h**), 9 (**c, f**) and 14 (**i**) days were stained with antibodies against human PHGR1 (green). The cells were counterstained with Alexa594-conjugated antibodies against the tight-junction protein occludin (red) and the DNA dye DAPI (blue). See Additional file 3 for negative isotype control staining of the Caco-2 cells. **a**-**c** Images taken with a 10× objective. Scale bars = 100 μm. **d**-**f** Images taken with a 100× objective. Scale bars = 10 μm. **g**-**i** Images taken with a confocal laser scanning microscope at 60× magnification. Scale bars = 10 μm. Z-X and Z-Y views are shown in the side panels

To determine the intracellular localization of PHGR1 protein during differentiation we cultured Caco-2 cells beyond confluence on optically transparent permeable filter inserts. The previously observed upregulation of *PHGR1* expression during Caco-2 cell differentiation was verified in the filter-grown cells by quantitative RT-PCR (Additional file 3). Immunofluorescent staining of PHGR1 protein revealed a primarily cytoplasmic localization in these differentiating cells (Fig. 4). Interestingly, immunofluorescent analyses revealed a striking heterogeneity in PHGR1 protein levels among the Caco-2 cells, especially in the early phase of differentiation. Some cells had high PHGR1 levels, but the majority had very low levels. The overall increase in PHGR1 protein levels during differentiation seemed to be caused by an increase in the fraction of Caco-2 cells expressing the PHGR1 protein, rather than by an increase in PHGR1 protein levels in cells already expressing the protein.

The cytosolic distribution of PHGR1 protein in Caco-2 cells at day 2 revealed an almost homogeneous distribution of tiny spots (Fig. 4d). Interestingly, the distribution shifted towards larger speckles of PHGR1 (Fig. 4f) inside the cytoplasm at day 9. From the focal plan we also judged the large speckles in later stages to be located on the apical side of the cells. Examination of the slides with a confocal laser scanning microscope confirmed this distribution (Fig. 4h and i). The speckles were reminiscent of intracellular vesicles.

### Transcript profile in response to PHGR1 downregulation

To elucidate the functional pathways related to PHGR1 expression, we investigated the global transcriptional effects of PHGR1 knockdown in HT29 colon cancer cells. The HT29 cancer cell line was chosen because of its moderately high *PHGR1* mRNA levels in growing cultures. To eliminate non-specific side effects of siRNA transfection, two different PHGR1-targeted siRNAs were used in addition to a negative control siRNA (scramble). Knockdown of *PHGR1* mRNA (70% reduction) and protein levels in transfected HT29 cells was verified by quantitative RT-PCR and Western blotting (Additional file 4).

**Fig. 5:**
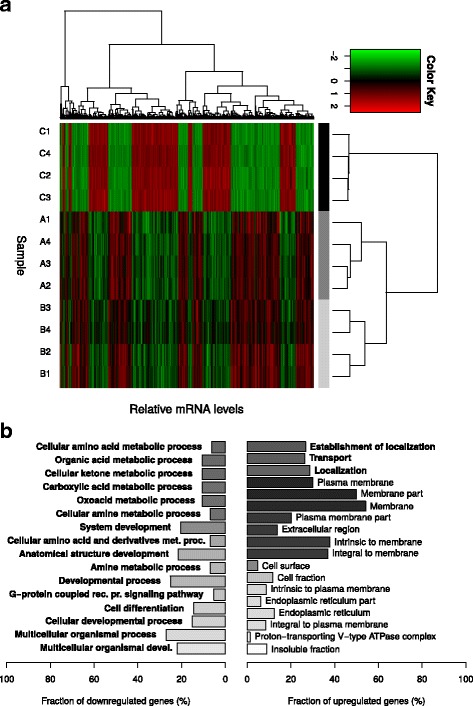
Transcript profiling of HT29 cells subjected to PHGR1 downregulation. **a** Hierarchical clustering of samples (horizontal) and mRNAs (vertical) in the global mRNA profile of HT29 cells subjected to PHGR1 knockdown. Only the mRNAs with significant changes in response to PHGR1 knockdown are shown. The color key is scaled for each mRNA (row) to visualize differences. The sample names are shown on the left side of the heatmap; letters denote siRNA (C = negative control siRNA, A and B = PHGR1-targeting siRNA A and B) and numbers denote biological replicates. **b** Gene ontology analysis of genes down- and upregulated in response to PHGR1 knockdown. Gene ontology terms significantly enriched after correction for multiple testing are listed, biological processes (GO_BP) in bold fonts and cellular compartments (GO_CC) in plain fonts. Bar lengths show the fraction of downregulated (left panel) and upregulated (right panel) genes associated with the specific gene ontology terms, whereas the bar color reflects the adjusted *P*-value for each term (white: *P* = 0.05, black: P → 0)

Global mRNA profiling by microarray analysis of HT29 cells transfected separately with two different PHGR1-targeted siRNAs (siRNA A and B) revealed 406 genes that were significantly upregulated and 369 genes significantly downregulated in response to PHGR1 knockdown (Additional files 5 and 6). Clustering and heatmap analysis of significantly upregulated or downregulated genes unveiled substantial changes in the mRNA profiles due to PHGR1 knockdown (Fig. 5a). The biological replicates clustered together as expected. Upregulated and downregulated transcripts with high fold changes or well-known functions were verified by quantitative RT-PCR in a separate transfection experiment (Additional file 4).

To further characterize the functional relationships of PHGR1, we performed gene ontology analysis, searching for ontologies over- and under-represented among the upregulated and downregulated genes. As shown in Fig. 5b and Additional file 7, genes associated with localization and transport were over-represented among the upregulated genes. On the other hand, genes associated with metabolic processes were over-represented among the downregulated genes, whereas metabolic and biosynthetic biological processes were under-represented among the upregulated genes and catabolic processes were under-represented among the downregulated genes (results not shown).

### PHGR1 is a potential marker for detection of lymph node metastasis in colorectal cancer

**Fig. 6:**
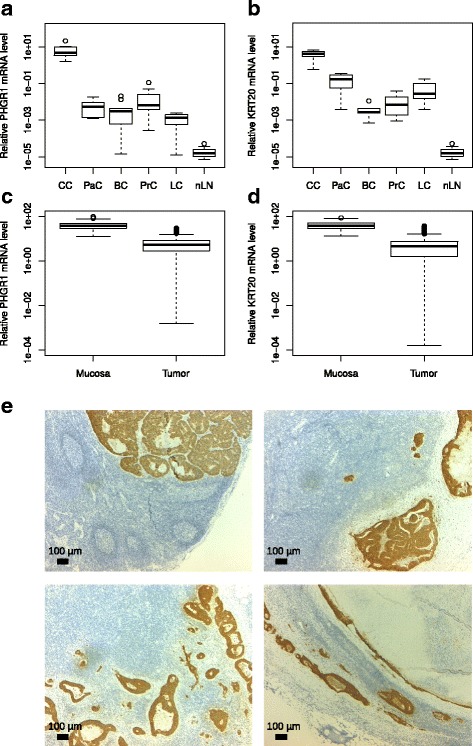
PHGR1 is a potential marker of lymph node metastasis. **a** Relative *PHGR1* mRNA levels in colon cancers (CC, *n* = 10), pancreatic cancers (PaC, *n* = 5), breast cancers (BC, *n* = 10), prostate cancers (PC, *n* = 10), non-small cell lung cancers (LC, *n* = 10), normal lymph nodes (nLN, *n* = 10), and lymph nodes with known colon cancer metastases (mLN, *n* = 10). **b** Relative *KRT20* mRNA levels in the same tissue samples as in (**a**). **c** Relative *PHGR1* mRNA levels in mucosa (*n* = 76) and tumor (*n* = 209) tissue samples from patients with operable colon cancer. **d** Relative *PHGR1* and *KRT20* mRNA levels in the same samples as in (**c**). **e** Immunohistochemical staining of PHGR1 protein in CRC lymph node metastases. Tissue sections from four different lymph nodes with known metastases were stained with PHGR1 antibody (brown) and nuclear counterstain (blue). The images were obtained with a 2.5× microscope objective

We hypothesized that PHGR1 may serve as a clinically useful biomarker for detection of lymph node metastasis in colorectal cancer patients because of our observation that *PHGR1* mRNA levels are high in normal colon mucosa and very low in normal lymph nodes and several other tissues (Fig. 2a). To investigate this hypothesis, we measured the levels of *PHGR1* mRNA in parallel with the mRNA levels of the established lymph node metastasis marker *KRT20* in tumors from colon, pancreas, breast, prostate and lung (non-small cell) cancer patients, as well as normal lymph nodes and lymph nodes with known colon cancer metastases (Fig. 6a and b). On average, *PHGR1* mRNA levels were 3.4 x 10^5^ times higher in colon tumors than normal lymph nodes, whereas *KRT20* mRNA levels were 1.3 × 10^5^ times higher (*P* = 0.02). Median *PHGR1* mRNA level were 1.4 × 10^5^ times higher in lymph nodes with known metastases compared with normal lymph nodes (*P* < 0.001), whereas the corresponding number was 2.5 x 10^4^ for *KRT20* (P < 0.001). This finding suggested that PHGR1 may be a promising alternative to KRT20 for detecting lymph node metastases. The mean *PHGR1* and *KRT20* mRNA levels in examined colon cancers were 620- and 90-times higher than the mean level in the other tumor types tested (*P* < 0.001), respectively, indicating that PHGR1 is also more colon cancer-specific than KRT20.

Downregulation during tumorigenesis is a recognized weakness of using differentiation markers such as KRT20 as markers of tumor metastasis. To compare PHGR1 and KRT20 in this context, we measured *PHGR1* and *KRT20* mRNA levels in tumor biopsies from 209 patients with operable colon cancer and normal mucosa biopsies from 76 of the 209 patients (Fig. 6c and d). The mean *PHGR1* and *KRT20* mRNA levels were 6.2 and 6.7-times lower in tumors than in the mucosa, respectively. No significant association was found between primary tumor *PHGR1* mRNA levels and clinicopathological parameters or survival (Additional file 8 and data not shown). PHGR1 protein was also detected by IHC in all examined colorectal tumors (*n* = 17, Fig. 3c and data not shown).

As a proof-of-principle experiment, we immunohistochemically stained the PHGR1 protein in several lymph nodes with known CRC metastases (Fig. 6e). All examined lymph nodes (*n* = 4) were clearly positive for PHGR1 in the metastatic areas, suggesting that the PHGR1 protein is a potential marker of lymph node metastasis in colorectal cancer.

## Discussion

Here, we report the first molecular characterization of the novel PHGR1 protein, which is specifically expressed in the gastrointestinal tract. PHGR1 is expressed primarily in the mature epithelial cells of the mucosal lining of the tract, is glycosylated and localizes in both the cytoplasm and nucleus. Transcript profiling and gene ontology analysis of HT29 cells subjected to PHGR1 knockdown revealed a functional relationship with transport and metabolic processes. Finally, we demonstrated that PHGR1 mRNA and protein could be promising biomarkers for the detection of lymph node metastases in colorectal cancer patients.

Homology searches often reveal structurally similar proteins that may shed light on the function of a novel protein. However, the small size and extraordinarily high proline, histidine, and glycine content of PHGR1 represented a challenge for this approach. Nonetheless, we found several apparent homologs when performing the searches without compositional adjustment. Thus, the similarities were based more on similar amino acid composition than actual sequence homology. Some of the proteins also had other interesting similarities with PHGR1. The proline-rich salivary proteins PRB2 and PRB3 are also glycosylated and have tandem repeats [19]. Several collagens are also proline-rich, repetitive and glycosylated [20]. Another family of small proline-rich proteins, the SPRRs, are crosslinked in a multi-protein complex of stratified squamous epithelium, called the cornified envelope [21]. These proteins are small (8–18 kDa) and harbor internal repeats of 8–12 amino acids, similar to PHGR1. Interestingly, in our study the genes encoding SPRR1B and SPRR3 were both significantly upregulated in response to PHGR1 downregulation (Additional file 5).

The multiple PHGR1 protein bands of unexpected size in our Western blot analysis encouraged us to affinity purify and validate our polyclonal PHGR1 antibody thoroughly. The strong correlation between protein and mRNA levels in multiple experiments (compare Figs. 2c and 3c, Fig. 3a and Additional file 2, and Fig. 3d and e) strengthened our perception that the PHGR1 antibody reaction was specific despite the unexpected results. Therefore, we searched other explanations for the unexpected band sizes, which were most likely due to some kind of post-translational modification of PHGR1. The modification had to be covalent because weaker bonds, such as disulfide bridges, are broken during SDS-PAGE. One alternative was isopeptide bonds generated by transglutaminases or ubiquitin/SUMO ligases [22, 23]. The single lysine residue of PHGR1 was the sole potential target for such modification. However, the resistance to trypsin digestion and the small differences in band sizes, corresponded to a modifier of flexible size and suggested glycosylation as a more likely explanation [24]. Intriguingly, the predicted PHGR1 peptide has very few potential glycosylation sites. The remaining alternative was hydroxylation and O-glycosylation of prolines or the single lysine residue. Glycosylation of hydroxyprolines has only been described for plants, whereas glycosylation of hydroxylysine occurs in collagens [20, 25]. The PHGR1 lysine being well conserved (Fig. 1) and having a glycine at the + 1 position, which is similar to the glycine required for collagen hydroxylation [20], supports O-glycosylation of this lysine residue in its potential hydroxylated form. TMFS acid treatment succeeded in removing the glycosylation, whereas enzymatic deglycosylation failed. This may be due to the complexity of O-glycosylation, which can block common enzymatic deglycosylation approaches [26].

The primary cytoplasmic distribution of PHGR1 protein in Caco-2 cells contrasted with the shift towards nuclear distribution observed in normal colonic mucosa cells (compare Figs. 4 and 3c). Interestingly, we also observed minor subclones of Caco-2 cells with PHGR1 present in both the nucleus and cytoplasm after 14 days of culture (Additional file 3). Full differentiation of all cells in a culture requires more time than full differentiation of minor clones [18], and the Caco-2 cells may therefore not have differentiated completely during our 14 day experiment. Varying culturing lengths have been reported for full differentiation of Caco-2 cells (from 14 to 30 days), possibly reflecting the heterogeneous nature of the cell line, which we also observed [18, 27, 28]. However, the biology of cancer cell lines is perturbed in various ways due to culturing, compared to the tissues from which they originate. Therefore, even if Caco-2 cells are generally accepted as a good model for intestinal enterocytes [18], our observations in this model should be interpreted with caution.

The speckled distribution of PHGR1 that we observed in differentiating Caco-2 cells (Fig. 4) suggested that PHGR1 may be associated with intracellular vesicles, such as endosomes [29, 30]. The resolution of the immunohistochemistry images in Fig. 3c was not sufficient to distinguish whether PHGR1 had a uniform or speckled distribution in the cytoplasm of normal enterocytes. Interestingly, the gene ontology analysis of genes significantly upregulated in response to PHGR1 knockdown revealed an over-representation of gene products localized to cellular membrane compartments (Fig. 5). Genes associated with transport were also significantly over-represented, suggesting that PHGR1 may be related to vesicle-mediated transport. We even found an over-representation of genes related to the proton−transporting vacuole-type ATPase complex found in the membrane of endosomes and other organelles [31]. The laser scanning microscope images indicated an apical, rather than basolateral, distribution of PHGR1 (Fig. 4g-i). Interestingly, evidence indicates that protein glycosylation plays a role in apical vesicle sorting, and that vesicle transport is important for maintenance of epithelial cell polarity [3, 32]. These observations encourage further research to elucidate the potential role of PHGR1 in vesicle-mediated transport.

The effects of knocking down a gene of interest on specific biological processes is difficult to interpret. The relationship between the gene of interest and the process may be indirect and the gene product may make a positive or a negative contribution to the process, neither of which can be unveiled by simple mRNA profiling.Therefore, the relationship between PHGR1 and metabolism and transport reported in our study is only hypothesis-generating. However, the transport aspect was supported by additional evidence (localization) and is also closely connected to the primary functions of intestinal epithelial cells: absorption and secretion [1].

Finally, we obtained evidence that both PHGR1 mRNA and protein may be promising biomarkers for the detection of metastases in colorectal cancer, as they have superior characteristics compared to the established metastasis marker KRT20. Differentiation markers (e.g. KRT20 and PHGR1) can be used to detect small deposits of tumor cells in clinical samples normally negative for such markers, such as lymph nodes from resection specimens [33]. This is valuable from a clinical point of view because metastases to regional lymph nodes is a well-known prognostic and predictive factor [34].

## Conclusions

We have here unveiled important pieces of the functional puzzle for the novel protein PHGR1. Our present evidence suggests a functional role in mature intestinal epithelial cells that is specifically related to vesicle-mediated transport. However, further research is required to complete the picture. We also expect medical researchers to explore the biomarker potential of PHGR1 in clinical studies, notably with regard to colorectal cancer.

## Additional files

Additional file 1: PHGR1 peptide sequence analysis. (PDF 31 kb)

Additional file 2: PHGR1 protein characterization. A) The barplot shows relative PHGR1 mRNA levels in the same samples that was analyzed in Fig. 3a, reproduced above. B) Immunohistochemical staining of PHGR1 in normal breast (1), liver (2), kidney (3), thyroid (4), pancreas (5) and placenta (6). (PDF 9130 kb)

Additional file 3: Immunofluorescent staining of PHGR1 protein in differentiating Caco-2 cells. (PDF 5310 kb)

Additional file 4: Verification experiments in relation to transcript profiling of HT29 cells subjected to PHGR1 downregulation. (PDF 901 kb)

Additional file 5: Upregulated genes. (PDF 82 kb)

Additional file 6: Downregulated genes. (PDF 79 kb)

Additional file 7: Gene ontology analysis. (PDF 41 kb)

Additional file 8: Clinicopathological data for the colorectal cancer patient cohort. (PDF 26 kb)

## Abbreviations

AP: Alkaline phosphatase
CGAP: Cancer Gene Anatomy Project
DGED: Digital gene expression displayer
DIG: Digoxigenin
EST: Expressed sequence tag
ORF: Open reading frame
PBS: Phosphate-buffered saline
PET: Polyethylene terephthalate
PHGR1: Proline-histidine-glycine rich 1
RLM-RACE: RNA ligase-mediated rapid amplification of 5’ cDNA ends
siRNA: Small interfering RNA
TFMS: Trifluoromethanesulfonic
