# Supplementary material for: Expression profiling and intracellular localization studies of the novel Proline-, Histidine-, and Glycine-rich protein 1 suggest an essential role in gastro-intestinal epithelium and a potential clinical application in colorectal cancer diagnostics
Source: BMC Gastroenterol. 2018 Feb 7;18:26. doi: 10.1186/s12876-018-0752-8 (PMC5803922; doi:10.1186/s12876-018-0752-8)
Supplement: Supplementary file 2 — PHGR1 protein characterization. A) The barplot shows relative PHGR1 mRNA levels in the same samples that was analyzed in Fig. 3a, reproduced above. B) Immunohistochemical staining of PHGR1 in normal breast (1), liver (2), kidney (3), thyroid (4), pancreas (5) and placenta (6). (PDF 9130 kb) [file 12876_2018_752_MOESM2_ESM.pdf]

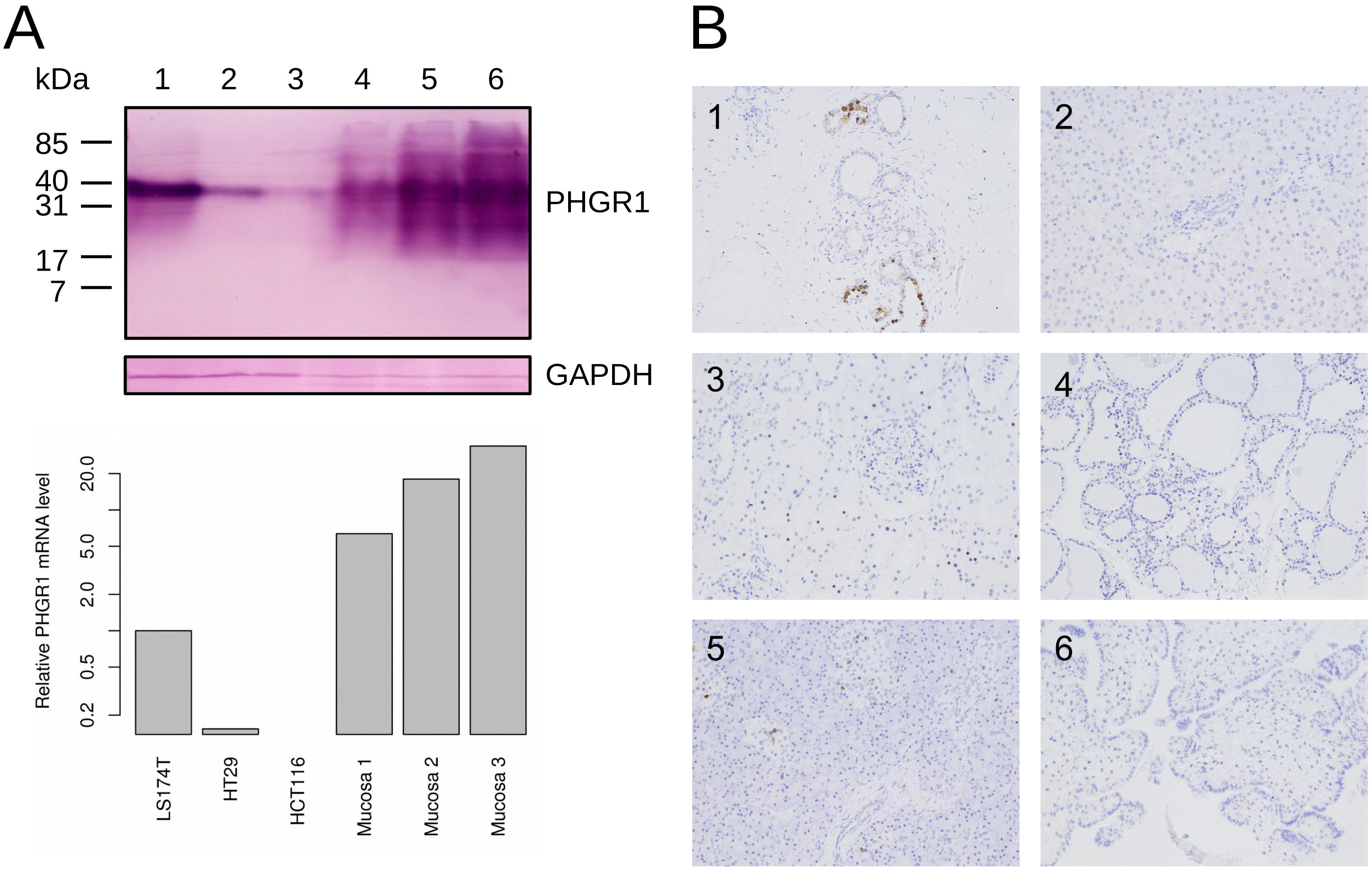

Additional file 2: PHGR1 protein characterization.

A) The barplot shows relative PHGR1 mRNA levels in the same samples that was analyzed in figure 3A, reproduced above.

B) Immunohistochemical staining of PHGR1 in normal breast (1), liver (2), kidney (3), thyroid (4), pancreas (5) and placenta (6).
