# Supplementary material for: Expression profiling and intracellular localization studies of the novel Proline-, Histidine-, and Glycine-rich protein 1 suggest an essential role in gastro-intestinal epithelium and a potential clinical application in colorectal cancer diagnostics
Source: BMC Gastroenterol. 2018 Feb 7;18:26. doi: 10.1186/s12876-018-0752-8 (PMC5803922; doi:10.1186/s12876-018-0752-8)
Supplement: Supplementary file 3 — Immunofluorescent staining of PHGR1 protein in differentiating Caco-2 cells. (PDF 5310 kb) [file 12876_2018_752_MOESM3_ESM.pdf]

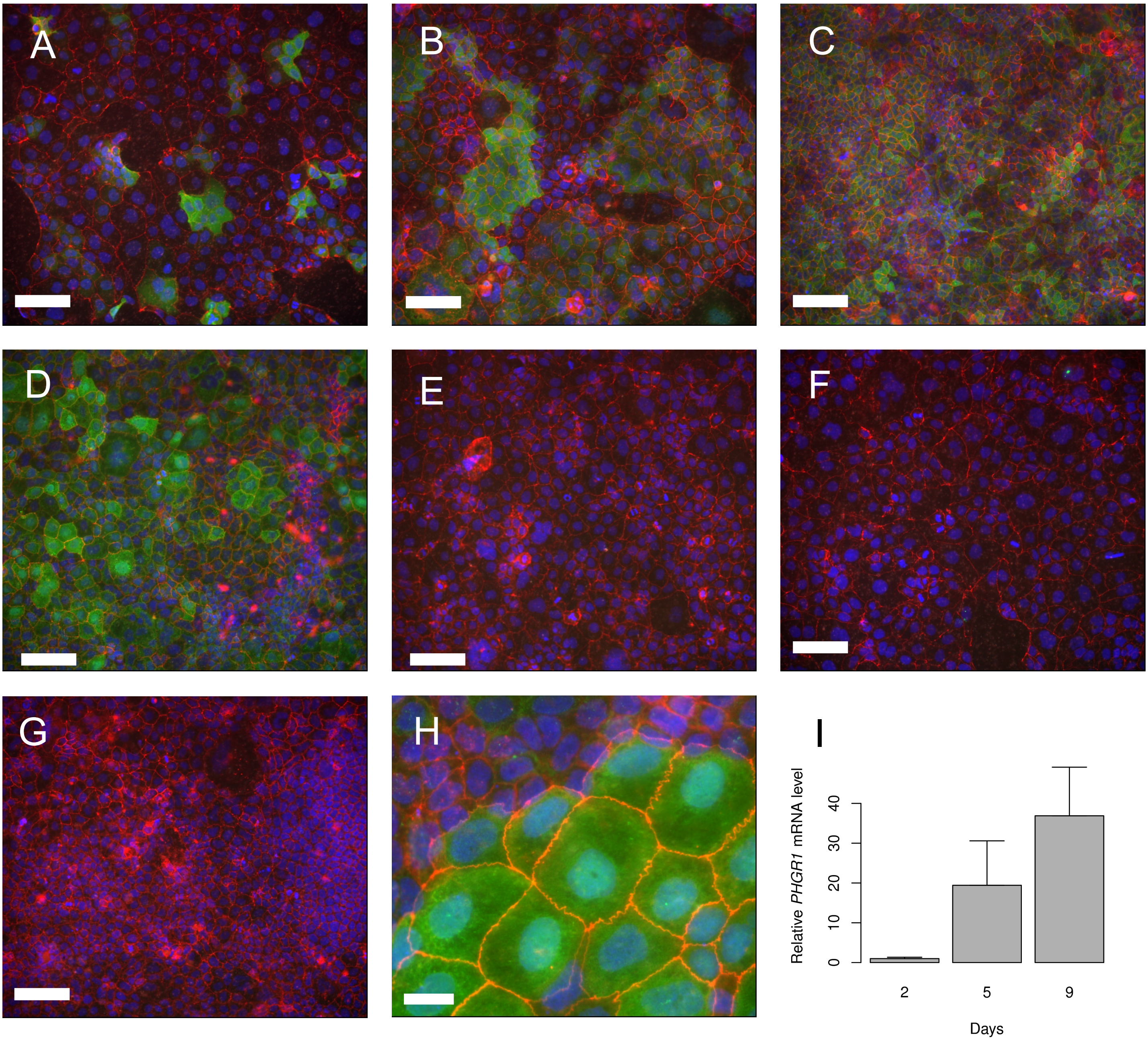

Additional file 3: Immunofluorescent staining of PHGR1 protein in differentiating Caco-2 cells.

A-H) Caco-2 cells were grown on filters for 2 (A,E), 5 (B,F), 9 (C) and 14 (D, G, H) days. The cells in panel A-D were stained with rabbit antibodies against human PHGR1, which were detected with a FITC-labelled secondary antibody (green). The cells in panel E-G were stained with a negative isotype control antibody and the same secondary antibody. All cells were counterstained with Alexa594-conjugated antibodies against the tight-junction protein occludin (red) and the DNA dye DAPI (blue). The white scale bars indicate 100 µm width in panel A-G, 10 µm in panel H.

I) Relative *PHGR1* mRNA level in differentiating Caco2 cells measured by quantitative reverse transcription PCR. The Caco-2 cells were cultured for 2, 5 and 9 days (control samples from the experiment in figure 1). The *PHGR1* levels were normalized against the *HPRT1* reference mRNA and day 2 levels. The error bars show standard deviation, estimated by error propagation.
