# Supplementary material for: Expression profiling and intracellular localization studies of the novel Proline-, Histidine-, and Glycine-rich protein 1 suggest an essential role in gastro-intestinal epithelium and a potential clinical application in colorectal cancer diagnostics
Source: BMC Gastroenterol. 2018 Feb 7;18:26. doi: 10.1186/s12876-018-0752-8 (PMC5803922; doi:10.1186/s12876-018-0752-8)
Supplement: Supplementary file 4 — Verification experiments in relation to transcript profiling of HT29 cells subjected to PHGR1 downregulation. (PDF 901 kb) [file 12876_2018_752_MOESM4_ESM.pdf]

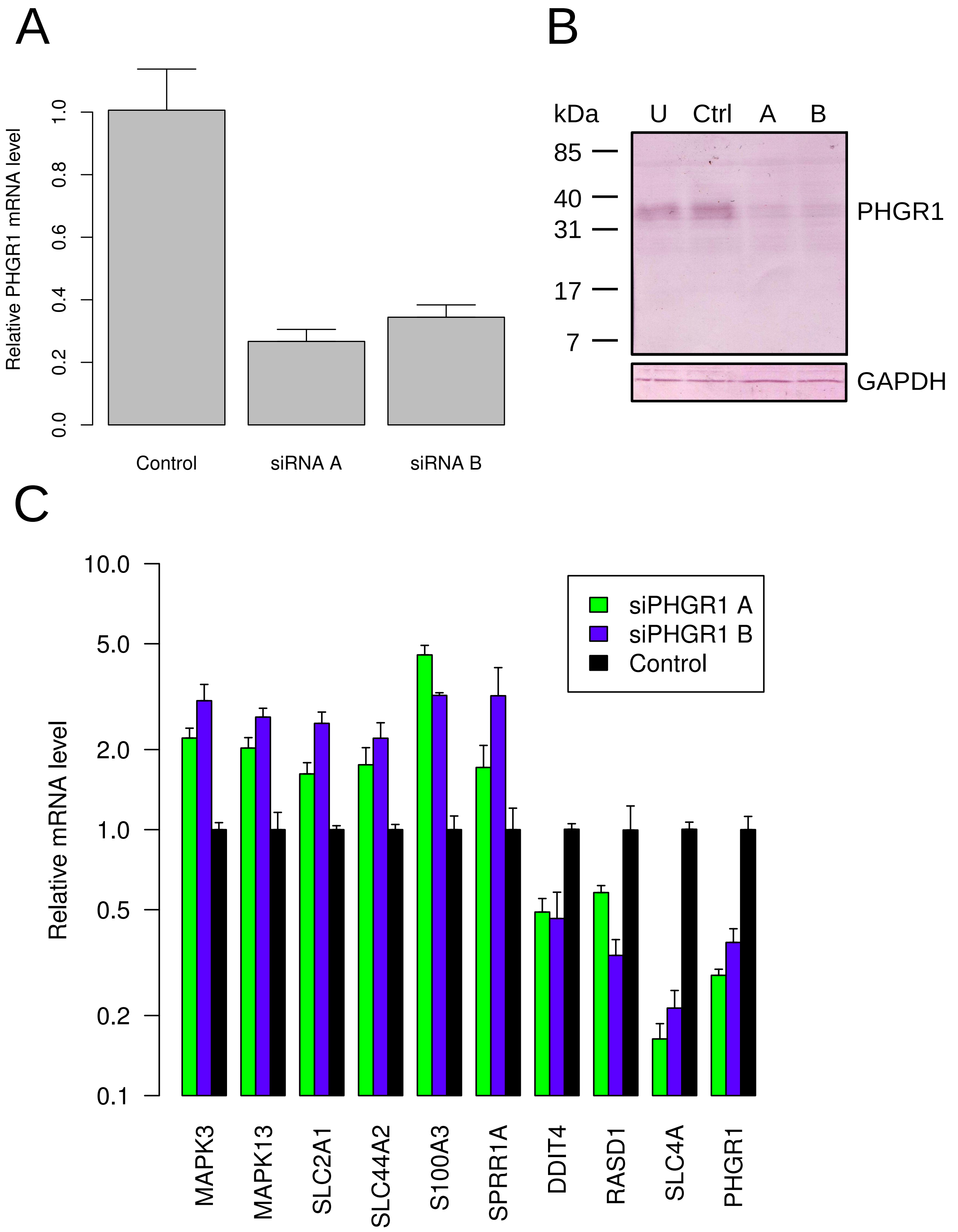

Additional file 4: Transcript profiling of HT29 cells subjected to PHGR1 downregulation.
A) Verification of PHGR1 mRNA knockdown by quantitative RT-PCR. HT29 cells were transfected with a negative control siRNA (Control) and two siRNAs targeted against PHGR1 mRNA (siRNA A and siRNA B). Relative PHGR1 mRNA levels were measured by quantiative RT-PCR, relative to a reference mRNA and the mean PHGR1 level in the control transfections. Mean levels are plotted, with error bars showing the standard deviation for the four biological replicates.
B) Verification of PHGR1 protein knockdown by Western blotting. Untransfected HT29 cells (U), HT29 cells transfected with a negative control siRNA (Ctrl) and two different siRNA targeting PHGR1 mRNA (A and B) were analyzed.
C) Verification of selected up- and downregulated transcripts in siRNA-transfected HT29 cells by quantitative RT-PCR. mRNA levels relative to a reference transcript and the mean of the control transfections are shown. Standard deviations from three bioloical replicates are shown by error bars.
