# Supplementary material for: Expression profiling and intracellular localization studies of the novel Proline-, Histidine-, and Glycine-rich protein 1 suggest an essential role in gastro-intestinal epithelium and a potential clinical application in colorectal cancer diagnostics
Source: BMC Gastroenterol. 2018 Feb 7;18:26. doi: 10.1186/s12876-018-0752-8 (PMC5803922; doi:10.1186/s12876-018-0752-8)
Supplement: Supplementary file 5 — Upregulated genes. (PDF 82 kb) [file 12876_2018_752_MOESM5_ESM.pdf]

Additional file 5: Upregulated genes

No	ILMN_Gene	Probe_ID	Score[1]	logFCmean[2]	adjPmean
1	LOC645553	ILMN_1676712	-1,84	-1,95	3,07E-10
2	LOC644760	ILMN_1661743	-1,50	-1,57	2,50E-08
3	HS.579631	ILMN_1881909	-1,36	-1,57	1,33E-05
4	LOC147645	ILMN_1733084	-1,28	-1,32	2,64E-09
5	EMP3	ILMN_1765446	-1,25	-1,53	4,71E-08
6	MGC59937	ILMN_1814106	-1,25	-1,36	1,97E-07
7	GDPD3	ILMN_1731870	-1,15	-1,47	1,06E-08
8	MAPK3	ILMN_1812747	-1,05	-1,25	1,49E-09
9	HSPA5	ILMN_1773865	-1,00	-1,09	3,04E-08
10	TCN1	ILMN_1768469	-0,98	-1,05	4,39E-08
11	DICER1	ILMN_1686725	-0,96	-0,96	5,64E-08
12	TMPRSS4	ILMN_1676727	-0,94	-1,04	2,31E-07
13	SGPL1	ILMN_1761531	-0,93	-0,98	4,94E-06
14	LOC205251	ILMN_1693685	-0,89	-1,02	8,62E-08
15	AKR1B1	ILMN_1701731	-0,88	-0,89	4,85E-08
16	ST3GAL4	ILMN_1750086	-0,86	-0,86	1,56E-06
17	TNNT1	ILMN_1717297	-0,85	-0,90	6,22E-06
18	MGC15523	ILMN_1759743	-0,84	-0,88	1,35E-06
19	ARL2	ILMN_1787879	-0,81	-1,06	5,71E-05
20	FAM65A	ILMN_1680037	-0,80	-0,81	1,66E-05
21	ACTR1A	ILMN_1792314	-0,79	-0,80	7,31E-06
22	SLC2A1	ILMN_1809256	-0,78	-0,96	2,30E-08
23	SLC22A18AS	ILMN_1691048	-0,77	-0,77	7,66E-08
24	ERP27	ILMN_1655261	-0,74	-0,79	2,63E-07
25	BAD	ILMN_1738652	-0,72	-0,93	2,73E-06
26	CASZ1	ILMN_1655191	-0,70	-0,99	8,36E-06
27	TINAGL1	ILMN_1807169	-0,70	-0,87	2,69E-05
28	TBC1D22B	ILMN_1723475	-0,69	-0,78	1,28E-07
29	RHOC	ILMN_1673305	-0,69	-0,73	1,42E-06
30	SLC44A2	ILMN_1771987	-0,69	-0,72	5,10E-06
31	TAF13	ILMN_1712561	-0,68	-0,71	4,09E-05
32	M-RIP	ILMN_1774547	-0,67	-0,80	6,80E-07
33	ARID3A	ILMN_1670130	-0,67	-0,80	9,14E-06
34	WFS1	ILMN_1759023	-0,67	-0,89	1,58E-04
35	NRBP1	ILMN_1670096	-0,67	-0,79	8,84E-07
36	LASS2	ILMN_1726108	-0,66	-0,67	1,02E-06
37	MTMR11	ILMN_1698246	-0,65	-0,74	1,13E-07
38	RNF149	ILMN_1665877	-0,63	-0,69	2,89E-07
39	SFTPG	ILMN_1703864	-0,63	-0,72	1,17E-05
40	TLN1	ILMN_1696643	-0,62	-0,68	2,32E-05
41	CBR3	ILMN_1652237	-0,62	-0,73	6,59E-07
42	IGFL2	ILMN_1745238	-0,62	-0,79	1,26E-05
43	CTSL1	ILMN_1812995	-0,62	-0,66	2,55E-06
44	SLC25A44	ILMN_1810514	-0,62	-0,65	8,81E-07
45	DPEP1	ILMN_1670779	-0,62	-0,63	6,89E-06
46	ARPC4	ILMN_1803183	-0,62	-0,65	1,24E-05

Additional file 5: Upregulated genes

47	CST1	ILMN_1753449	-0,61	-0,74	2,68E-07
48	VAMP5	ILMN_1809467	-0,61	-0,64	3,87E-06
49	PRSS8	ILMN_1796461	-0,61	-0,93	5,13E-05
50	AIM1L	ILMN_1669557	-0,60	-0,64	1,16E-06
51	C11ORF9	ILMN_1811437	-0,60	-0,65	6,67E-05
52	TSPAN4	ILMN_1720908	-0,60	-0,60	3,43E-04
53	RFFL	ILMN_1678010	-0,60	-0,65	1,76E-06
54	NCKAP1	ILMN_1747392	-0,60	-0,73	3,07E-05
55	SH3GL1	ILMN_1788062	-0,60	-0,75	3,24E-05
56	PARD6B	ILMN_1745154	-0,60	-0,62	3,97E-05
57	COMT	ILMN_1810941	-0,59	-0,60	7,15E-05
58	BTBD16	ILMN_1677684	-0,59	-0,68	1,13E-06
59	SNX17	ILMN_1732810	-0,59	-0,77	3,09E-06
60	SLC46A3	ILMN_1658639	-0,59	-0,70	6,78E-07
61	CLPTM1	ILMN_1665831	-0,59	-0,62	2,06E-06
62	KLK11	ILMN_1695924	-0,59	-0,62	3,72E-05
63	RIPK3	ILMN_1763763	-0,59	-0,65	4,96E-06
64	RPPH1	ILMN_1704056	-0,58	-0,70	8,47E-07
65	SIRT7	ILMN_1800418	-0,58	-0,74	1,71E-04
66	CRB3	ILMN_1754635	-0,58	-0,60	1,09E-04
67	KIAA1539	ILMN_1732609	-0,58	-0,72	4,44E-05
68	LRP8	ILMN_1677765	-0,57	-0,62	6,18E-05
69	HS.137274	ILMN_1892548	-0,56	-0,61	6,59E-06
70	POLR3F	ILMN_1673966	-0,56	-0,63	1,04E-06
71	LHFPL2	ILMN_1811077	-0,56	-0,57	7,80E-06
72	RASSF3	ILMN_1761026	-0,56	-0,62	1,99E-04
73	ISCU	ILMN_1735432	-0,56	-0,61	4,97E-06
74	KLK3	ILMN_1655426	-0,55	-0,71	6,00E-06
75	TMEM120A	ILMN_1654516	-0,55	-0,58	7,86E-05
76	OKL38	ILMN_1795963	-0,55	-0,63	1,68E-04
77	CLIC1	ILMN_1716360	-0,55	-0,66	1,28E-04
78	KLK6	ILMN_1777541	-0,55	-0,66	1,37E-05
79	DHX32	ILMN_1713688	-0,55	-0,56	1,36E-04
80	TBX10	ILMN_1682979	-0,55	-0,72	1,02E-05
81	C19ORF6	ILMN_1762439	-0,55	-0,59	1,32E-05
82	FAM125A	ILMN_1664587	-0,55	-0,78	1,32E-06
83	LOC643722	ILMN_1652649	-0,54	-0,69	2,35E-05
84	MBOAT5	ILMN_1805225	-0,54	-0,55	1,43E-05
85	S100A3	ILMN_1712545	-0,54	-1,23	7,42E-07
86	S100A2	ILMN_1725852	-0,54	-0,62	6,24E-06
87	A4GALT	ILMN_1735045	-0,54	-0,57	2,13E-05
88	ISG20	ILMN_1659913	-0,54	-0,99	6,45E-07
89	MAP7D1	ILMN_1733348	-0,54	-0,60	7,13E-06
90	ELF4	ILMN_1652082	-0,54	-0,81	1,15E-05
91	BCAR1	ILMN_1672596	-0,54	-0,71	1,17E-05
92	CTDSP2	ILMN_1692962	-0,54	-0,58	1,09E-05
93	RAB10	ILMN_1793433	-0,53	-0,54	1,00E-05

Additional file 5: Upregulated genes

94	NEU1	ILMN_1763144	-0,53	-0,76	8,09E-06
95	ATP6V0E1	ILMN_1715635	-0,53	-0,59	8,80E-06
96	DYNC1LI2	ILMN_1783448	-0,53	-0,76	1,83E-06
97	FAM127B	ILMN_1686254	-0,53	-0,58	2,95E-06
98	DNM3	ILMN_1680928	-0,53	-0,98	2,62E-07
99	C17ORF60	ILMN_1747347	-0,52	-0,57	1,70E-06
100	IDH1	ILMN_1696432	-0,52	-0,56	1,75E-05
101	KCTD20	ILMN_1803476	-0,52	-0,68	6,96E-06
102	HS.163264	ILMN_1874613	-0,52	-0,80	2,14E-05
103	ADFP	ILMN_1660332	-0,52	-0,72	4,04E-07
104	DCBLD2	ILMN_1735499	-0,52	-1,06	1,05E-07
105	C20ORF54	ILMN_1740923	-0,52	-0,65	1,31E-05
106	MAPK13	ILMN_1749327	-0,52	-0,76	4,82E-05
107	RALA	ILMN_1755364	-0,52	-0,59	5,31E-06
108	MORN2	ILMN_1756826	-0,51	-0,55	1,05E-04
109	APP	ILMN_1678152	-0,51	-0,58	3,14E-06
110	MAPRE3	ILMN_1734290	-0,51	-0,68	3,12E-05
111	CD68	ILMN_1714861	-0,51	-0,63	7,06E-06
112	KIAA1949	ILMN_1732967	-0,51	-0,53	7,28E-05
113	SENP5	ILMN_1675501	-0,50	-0,65	9,67E-07
114	GALR2	ILMN_1733847	-0,50	-0,69	2,74E-05
115	SERPINA5	ILMN_1759910	-0,50	-0,54	6,99E-05
116	NR1H2	ILMN_1691345	-0,50	-0,67	1,73E-05
117	CDS1	ILMN_1801476	-0,50	-0,53	7,99E-06
118	SF3B14	ILMN_1703720	-0,49	-0,57	1,59E-05
119	CDC42EP2	ILMN_1652777	-0,49	-0,60	6,08E-06
120	LOC652183	ILMN_1778301	-0,49	-0,56	1,55E-05
121	PEX26	ILMN_1738065	-0,49	-0,64	8,35E-05
122	MGC14376	ILMN_1660841	-0,49	-0,88	3,06E-06
123	MTMR12	ILMN_1802831	-0,49	-0,72	2,07E-04
124	TMEM62	ILMN_1745807	-0,49	-0,49	1,78E-05
125	BACE2	ILMN_1669323	-0,49	-0,51	1,60E-04
126	CDCP1	ILMN_1724941	-0,49	-0,55	1,73E-05
127	RSPRY1	ILMN_1763694	-0,49	-0,49	8,56E-05
128	EIF5A2	ILMN_1735151	-0,49	-0,55	2,29E-05
129	SLC5A6	ILMN_1741054	-0,48	-0,71	1,47E-05
130	CORO1A	ILMN_1713749	-0,48	-0,61	5,63E-06
131	TMEM127	ILMN_1721563	-0,48	-0,66	2,31E-05
132	DCPS	ILMN_1740737	-0,48	-0,83	3,66E-05
133	ATP6V0B	ILMN_1721391	-0,48	-0,50	9,98E-05
134	STX3	ILMN_1659544	-0,48	-0,64	9,24E-06
135	RNF24	ILMN_1717809	-0,48	-0,73	2,35E-04
136	PLP2	ILMN_1738767	-0,48	-0,57	1,51E-05
137	NUFIP2	ILMN_1765829	-0,47	-0,52	3,65E-04
138	CDK6	ILMN_1802615	-0,47	-0,58	3,31E-05
139	SERTAD3	ILMN_1813955	-0,47	-0,60	2,75E-05
140	CD163L1	ILMN_1661905	-0,47	-0,56	1,77E-05

Additional file 5: Upregulated genes

141	CXCL16	ILMN_1672278	-0,47	-0,71	5,46E-06
142	DNAJC5	ILMN_1719709	-0,47	-0,57	3,37E-04
143	CINP	ILMN_1765257	-0,47	-0,59	1,06E-05
144	SNX11	ILMN_1696051	-0,47	-0,49	3,21E-05
145	FNBP1L	ILMN_1754600	-0,47	-0,77	7,04E-05
146	CALR	ILMN_1736256	-0,47	-0,47	3,26E-04
147	ADIPOR2	ILMN_1750651	-0,47	-0,63	4,52E-06
148	UGT8	ILMN_1758816	-0,46	-0,53	2,14E-05
149	TRIM29	ILMN_1657766	-0,46	-0,50	5,67E-05
150	LOC648245	ILMN_1791141	-0,46	-0,55	4,59E-04
151	ACSS2	ILMN_1675125	-0,46	-0,77	1,35E-06
152	PEX5	ILMN_1660232	-0,46	-0,56	1,12E-05
153	SHB	ILMN_1732612	-0,46	-0,52	1,70E-04
154	C21ORF25	ILMN_1652512	-0,46	-0,63	1,08E-05
155	OSTM1	ILMN_1720303	-0,46	-0,65	3,77E-05
156	PHLDA2	ILMN_1671557	-0,45	-0,60	9,49E-05
157	HS.534682	ILMN_1892086	-0,45	-0,75	4,53E-05
158	UHMK1	ILMN_1702513	-0,45	-0,61	3,43E-04
159	MRPS7	ILMN_1813389	-0,45	-0,57	1,13E-04
160	PDLIM7	ILMN_1814985	-0,45	-0,58	1,20E-04
161	AP1S2	ILMN_1766411	-0,45	-0,64	6,22E-06
162	EIF2B1	ILMN_1753716	-0,44	-0,51	8,00E-06
163	TRAM2	ILMN_1788783	-0,44	-0,57	4,15E-06
164	CDA	ILMN_1714592	-0,44	-1,04	8,83E-07
165	PIM1	ILMN_1809933	-0,44	-0,54	1,69E-05
166	RTN4R	ILMN_1737484	-0,44	-0,58	1,37E-04
167	GNB1L	ILMN_1761044	-0,44	-0,47	9,02E-05
168	OTUD6B	ILMN_1772703	-0,43	-0,49	1,21E-05
169	PABPN1	ILMN_1759154	-0,43	-0,59	3,38E-04
170	SCD	ILMN_1689329	-0,43	-0,52	1,40E-05
171	YOD1	ILMN_1678919	-0,43	-0,51	1,43E-04
172	GUCA2A	ILMN_1763749	-0,43	-0,46	2,00E-05
173	PSMD11	ILMN_1800952	-0,43	-0,56	7,27E-05
174	SDF2L1	ILMN_1749213	-0,43	-0,59	2,07E-04
175	FHL3	ILMN_1703558	-0,43	-0,47	1,79E-05
176	HECTD3	ILMN_1813731	-0,43	-0,50	5,59E-05
177	LOC728825	ILMN_1667970	-0,43	-0,57	1,37E-04
178	MGC52282	ILMN_1770765	-0,43	-0,77	9,91E-06
179	SLC16A4	ILMN_1804673	-0,43	-0,59	6,48E-06
180	GPR115	ILMN_1684653	-0,43	-0,55	1,06E-05
181	LRRC8A	ILMN_1739840	-0,43	-0,48	1,50E-05
182	TIMP1	ILMN_1711566	-0,43	-0,60	4,13E-06
183	RNASE1	ILMN_1695339	-0,42	-0,48	1,35E-05
184	SNX8	ILMN_1804051	-0,42	-0,57	4,16E-05
185	LOC653157	ILMN_1665332	-0,42	-0,69	3,17E-05
186	ATP6V1F	ILMN_1678308	-0,42	-0,56	3,06E-04
187	MRPL50	ILMN_1664833	-0,42	-0,44	2,35E-04

Additional file 5: Upregulated genes

188	AP1M1	ILMN_1754738	-0,42	-0,51	4,98E-04
189	IL10RB	ILMN_1767360	-0,42	-0,44	4,23E-05
190	MVP	ILMN_1803277	-0,42	-0,62	1,33E-05
191	ZNF395	ILMN_1772876	-0,42	-0,45	6,47E-05
192	LOC390466	ILMN_1665781	-0,41	-0,61	3,20E-05
193	SCNN1A	ILMN_1713995	-0,41	-0,46	2,69E-04
194	ZDHHC9	ILMN_1733284	-0,41	-0,78	2,62E-07
195	LOC652489	ILMN_1666564	-0,41	-0,56	6,85E-05
196	LOC201175	ILMN_1656361	-0,41	-0,73	8,71E-05
197	HOXA10	ILMN_1682110	-0,41	-0,50	6,40E-05
198	LRPAP1	ILMN_1660341	-0,41	-0,60	6,05E-05
199	PARP16	ILMN_1812080	-0,41	-0,45	3,40E-05
200	ATP6V0D1	ILMN_1795826	-0,40	-0,51	2,29E-05
201	MEF2D	ILMN_1763228	-0,40	-0,48	1,60E-04
202	PHCA	ILMN_1812552	-0,40	-0,55	4,34E-04
203	ITPKA	ILMN_1776516	-0,40	-0,60	1,26E-05
204	LOC124220	ILMN_1742830	-0,40	-0,59	1,28E-04
205	PLAUR	ILMN_1666507	-0,40	-0,71	1,69E-06
206	WDR55	ILMN_1678957	-0,40	-0,40	1,14E-04
207	LASP1	ILMN_1665909	-0,40	-0,44	4,06E-05
208	C19ORF24	ILMN_1678052	-0,40	-0,53	1,11E-04
209	TMEM8	ILMN_1741371	-0,40	-0,50	3,20E-05
210	SLC39A14	ILMN_1764629	-0,40	-0,46	2,10E-05
211	GPRC5A	ILMN_1682599	-0,40	-0,56	2,30E-05
212	CTSB	ILMN_1696360	-0,39	-0,50	1,47E-05
213	SRPR	ILMN_1785660	-0,39	-0,46	3,31E-05
214	CYB561	ILMN_1679721	-0,39	-0,57	5,61E-06
215	C9ORF89	ILMN_1659189	-0,39	-0,56	1,26E-04
216	F3	ILMN_1797009	-0,39	-0,63	1,91E-06
217	TBPL1	ILMN_1708147	-0,39	-0,50	3,98E-05
218	RAB40B	ILMN_1685820	-0,39	-0,40	1,22E-04
219	CALM1	ILMN_1778242	-0,38	-0,48	7,37E-06
220	PCTP	ILMN_1802257	-0,38	-0,51	1,48E-05
221	ATG9A	ILMN_1739008	-0,38	-0,41	2,48E-04
222	C14ORF147	ILMN_1699676	-0,38	-0,52	8,30E-06
223	TMEM184B	ILMN_1747460	-0,38	-0,70	2,07E-04
224	WDR26	ILMN_1719343	-0,38	-0,41	9,02E-05
225	LOC728910	ILMN_1658569	-0,38	-0,59	2,63E-05
226	BCL2L12	ILMN_1731193	-0,38	-0,40	1,39E-04
227	TRIM16	ILMN_1680487	-0,37	-0,47	2,46E-04
228	BTBD14A	ILMN_1809522	-0,37	-0,53	1,15E-04
229	SIDT2	ILMN_1791912	-0,37	-0,48	3,17E-05
230	NBEAL2	ILMN_1660629	-0,37	-0,45	2,69E-04
231	PHC2	ILMN_1808047	-0,37	-0,44	4,00E-05
232	LEMD2	ILMN_1680860	-0,37	-0,39	3,45E-04
233	UPP1	ILMN_1798256	-0,37	-0,67	1,90E-05
234	M6PRBP1	ILMN_1660021	-0,37	-0,44	3,06E-05

Additional file 5: Upregulated genes

235	LOC728229	ILMN_1785919	-0,37	-0,37	2,32E-04
236	DERL2	ILMN_1761969	-0,37	-0,38	3,50E-04
237	GPATCH4	ILMN_1694837	-0,37	-0,41	2,63E-04
238	SPRR1A	ILMN_1716591	-0,36	-0,54	7,23E-05
239	PLOD3	ILMN_1714350	-0,36	-0,44	6,60E-05
240	SSNA1	ILMN_1715705	-0,36	-0,45	1,12E-04
241	TGFBR2	ILMN_1726245	-0,36	-0,57	1,73E-05
242	SULT1A3	ILMN_1815283	-0,36	-0,39	1,70E-04
243	HOXB7	ILMN_1702125	-0,36	-0,41	1,02E-04
244	DIRC2	ILMN_1793743	-0,36	-0,63	2,40E-05
245	TRIM15	ILMN_1711197	-0,36	-0,54	4,76E-05
246	EVI5	ILMN_1746314	-0,36	-0,37	3,31E-04
247	TICAM1	ILMN_1815079	-0,36	-0,39	1,74E-04
248	MACROD1	ILMN_1740960	-0,36	-0,41	1,40E-04
249	CCND3	ILMN_1668721	-0,36	-0,63	4,02E-04
250	KRT19	ILMN_1753924	-0,35	-0,39	6,04E-05
251	GPR37	ILMN_1668271	-0,35	-0,46	1,77E-04
252	SLC2A4RG	ILMN_1740430	-0,35	-0,41	5,97E-05
253	HS.574530	ILMN_1896129	-0,35	-0,35	3,09E-04
254	RHBDF1	ILMN_1808404	-0,35	-0,46	1,28E-04
255	SLC9A6	ILMN_1677829	-0,35	-0,42	2,02E-04
256	NOS3	ILMN_1775224	-0,35	-0,65	1,66E-04
257	ARMC7	ILMN_1797298	-0,35	-0,40	2,99E-04
258	CHMP4A	ILMN_1702828	-0,35	-0,40	3,67E-04
259	C1ORF210	ILMN_1670064	-0,35	-0,40	2,00E-04
260	NP	ILMN_1777534	-0,35	-0,52	1,05E-04
261	ALS2	ILMN_1750256	-0,35	-0,42	5,54E-05
262	SLC7A6	ILMN_1812559	-0,35	-0,69	4,94E-05
263	DMAP1	ILMN_1773885	-0,35	-0,42	1,49E-04
264	DEFB1	ILMN_1686573	-0,35	-0,68	9,80E-05
265	LOC643357	ILMN_1760338	-0,34	-0,42	3,80E-04
266	PXN	ILMN_1684440	-0,34	-0,47	5,73E-05
267	ANKRD13A	ILMN_1689908	-0,34	-0,49	3,61E-05
268	SMG7	ILMN_1690469	-0,34	-0,48	1,77E-04
269	SAV1	ILMN_1789552	-0,34	-0,45	1,47E-04
270	CYB5A	ILMN_1714167	-0,34	-0,58	8,50E-05
271	ADAM8	ILMN_1708348	-0,34	-0,52	1,56E-04
272	SLC22A5	ILMN_1699357	-0,34	-0,34	2,30E-04
273	SYNJ2	ILMN_1751749	-0,34	-0,40	3,44E-04
274	KUA-UEV	ILMN_1677446	-0,34	-0,42	2,63E-04
275	EHD1	ILMN_1651832	-0,33	-0,35	3,22E-04
276	RHPN2	ILMN_1753143	-0,33	-0,40	7,61E-05
277	BCL9L	ILMN_1743966	-0,33	-0,58	3,43E-04
278	DYNLL2	ILMN_1772796	-0,33	-0,35	2,63E-04
279	PVR	ILMN_1677305	-0,33	-0,35	6,51E-04
280	NEDD4	ILMN_1807881	-0,33	-0,35	3,59E-04
281	PTPRE	ILMN_1660907	-0,33	-0,36	1,47E-04

Additional file 5: Upregulated genes

282	GPATCH3	ILMN_1686929	-0,33	-0,37	4,10E-04
283	FLJ20186	ILMN_1767509	-0,33	-0,37	1,11E-04
284	KRT80	ILMN_1705814	-0,33	-0,50	4,83E-05
285	KCTD10	ILMN_1719064	-0,33	-0,38	1,79E-04
286	TTC26	ILMN_1676555	-0,33	-0,36	2,39E-04
287	APOBEC1	ILMN_1813881	-0,33	-0,42	1,51E-04
288	HS.558072	ILMN_1861057	-0,33	-0,43	6,36E-05
289	PCSK6	ILMN_1676595	-0,33	-0,36	5,34E-05
290	MARK2	ILMN_1789692	-0,33	-0,35	2,26E-04
291	SULT2B1	ILMN_1763520	-0,33	-0,78	4,32E-06
292	TM4SF1	ILMN_1770338	-0,33	-0,36	2,32E-04
293	MYLIP	ILMN_1656111	-0,33	-0,61	1,22E-04
294	HS.348844	ILMN_1915188	-0,33	-0,41	1,44E-04
295	HS.200085	ILMN_1820327	-0,32	-0,33	3,65E-04
296	CSNK1D	ILMN_1720708	-0,32	-0,43	3,35E-05
297	SC4MOL	ILMN_1720889	-0,32	-0,51	1,94E-04
298	IRF3	ILMN_1765649	-0,32	-0,40	2,00E-04
299	RAB5B	ILMN_1752582	-0,32	-0,43	8,83E-05
300	C13ORF15	ILMN_1658494	-0,32	-0,49	1,62E-04
301	MFSD5	ILMN_1702065	-0,32	-0,43	1,36E-04
302	C1ORF21	ILMN_1797735	-0,32	-0,45	3,93E-04
303	NTSR1	ILMN_1778831	-0,32	-0,54	1,25E-04
304	EFNA1	ILMN_1775903	-0,32	-0,45	4,86E-05
305	UBE2Q1	ILMN_1811751	-0,32	-0,36	4,39E-04
306	MAP1A	ILMN_1772366	-0,32	-0,37	5,22E-04
307	STAM	ILMN_1765409	-0,32	-0,38	2,03E-04
308	MGC57346	ILMN_1784428	-0,32	-0,73	8,81E-05
309	RNF11	ILMN_1810785	-0,32	-0,39	3,32E-05
310	CTNS	ILMN_1807719	-0,31	-0,41	2,17E-04
311	UBTD1	ILMN_1794914	-0,31	-0,45	2,32E-04
312	PHLDA3	ILMN_1659106	-0,31	-0,64	7,70E-05
313	HMGCS1	ILMN_1797728	-0,31	-0,45	2,08E-04
314	ZFAND2A	ILMN_1694671	-0,31	-0,55	1,55E-04
315	LAT	ILMN_1658322	-0,31	-0,34	2,01E-04
316	SLC36A1	ILMN_1787826	-0,31	-0,78	4,51E-05
317	MLLT11	ILMN_1759097	-0,31	-0,58	1,29E-04
318	CLTB	ILMN_1709346	-0,31	-0,42	9,04E-05
319	SLC24A6	ILMN_1701655	-0,30	-0,57	1,53E-04
320	CDKN1A	ILMN_1787212	-0,30	-0,97	1,07E-06
321	TMED9	ILMN_1743655	-0,30	-0,38	1,36E-04
322	SPRR3	ILMN_1810835	-0,30	-0,54	3,66E-05
323	C11ORF59	ILMN_1815878	-0,30	-0,34	4,31E-04
324	MAG1	ILMN_1794875	-0,30	-0,87	6,71E-06
325	BTBD7	ILMN_1757298	-0,30	-0,32	2,30E-04
326	RPL41	ILMN_1710001	-0,30	-0,41	2,03E-04
327	MOSPD1	ILMN_1765276	-0,29	-0,44	2,67E-05
328	MCOLN1	ILMN_1764383	-0,29	-0,39	5,00E-04

Additional file 5: Upregulated genes

329	RPA2	ILMN_1698510	-0,29	-0,37	4,50E-04
330	ELOVL6	ILMN_1700546	-0,29	-0,45	1,68E-04
331	DUSP5	ILMN_1656501	-0,29	-0,84	6,17E-06
332	HERC5	ILMN_1729749	-0,29	-0,50	1,10E-04
333	ARMET	ILMN_1682514	-0,29	-0,42	1,78E-04
334	FKSG30	ILMN_1814998	-0,29	-0,39	3,67E-04
335	CYP24A1	ILMN_1685663	-0,29	-0,36	5,05E-04
336	CYP39A1	ILMN_1680417	-0,28	-0,30	3,66E-04
337	MOBKL2C	ILMN_1719677	-0,28	-0,35	4,34E-04
338	CRIM1	ILMN_1809793	-0,28	-0,32	2,84E-04
339	SLC16A3	ILMN_1808982	-0,28	-0,58	2,36E-04
340	CMTM7	ILMN_1698934	-0,28	-0,71	4,71E-05
341	CLDN4	ILMN_1751764	-0,28	-0,30	6,35E-04
342	S100A4	ILMN_1688780	-0,28	-0,50	2,10E-04
343	SSFA2	ILMN_1742260	-0,28	-0,44	3,88E-04
344	FLVCR1	ILMN_1661596	-0,28	-0,35	4,76E-04
345	HS.538259	ILMN_1838313	-0,28	-0,49	3,51E-04
346	NNMT	ILMN_1715508	-0,28	-0,43	2,56E-04
347	CDC25A	ILMN_1733396	-0,27	-0,46	1,55E-04
348	D15WSU75E	ILMN_1737580	-0,27	-0,40	3,57E-04
349	ADM	ILMN_1708934	-0,27	-0,46	2,48E-04
350	LOC728760	ILMN_1658563	-0,27	-0,35	2,84E-04
351	CLN5	ILMN_1778203	-0,27	-0,44	5,12E-04
352	SSX2IP	ILMN_1720844	-0,27	-0,61	2,57E-04
353	LRRC51	ILMN_1685836	-0,27	-0,46	1,16E-04
354	KRT81	ILMN_1801442	-0,26	-0,52	3,14E-04
355	TMEM79	ILMN_1656312	-0,26	-0,54	3,29E-04
356	DAB2	ILMN_1764228	-0,26	-0,62	8,42E-05
357	KISS1	ILMN_1669404	-0,26	-0,82	3,85E-04
358	MVK	ILMN_1786310	-0,25	-0,29	6,66E-04
359	AXUD1	ILMN_1703123	-0,25	-0,60	3,78E-04
360	FANCG	ILMN_1744900	-0,25	-0,38	6,92E-04
361	C11ORF24	ILMN_1808163	-0,25	-0,34	2,94E-04
362	HS.573047	ILMN_1850816	-0,25	-0,43	2,57E-04
363	C20ORF24	ILMN_1679195	-0,25	-0,29	7,23E-04
364	TSC22D4	ILMN_1706609	-0,24	-0,38	4,23E-04
365	ATF5	ILMN_1669113	-0,24	-0,36	4,45E-04
366	CLIC3	ILMN_1796423	-0,24	-0,38	2,06E-04
367	LOC653888	ILMN_1804530	-0,24	-0,42	4,44E-04
368	MICA	ILMN_1655675	-0,24	-0,44	4,70E-04
369	TEAD2	ILMN_1682781	-0,23	-0,62	2,12E-04
370	RBM35A	ILMN_1749180	-0,23	-0,37	4,48E-04
371	CDC34	ILMN_1713006	-0,23	-0,60	2,96E-04
372	ARRB1	ILMN_1735218	-0,23	-0,28	5,01E-04
373	NFKBIA	ILMN_1773154	-0,23	-0,62	7,33E-05
374	TNFRSF10D	ILMN_1666022	-0,23	-0,36	3,09E-04
375	B3GALTL	ILMN_1764813	-0,23	-0,42	4,78E-04

Additional file 5: Upregulated genes

376	CLIP4	ILMN_1759792	-0,23	-0,67	5,53E-05
377	TRIM8	ILMN_1746704	-0,23	-0,40	1,19E-04
378	CSTB	ILMN_1761797	-0,23	-0,32	4,27E-04
379	PNPLA7	ILMN_1662587	-0,22	-0,59	4,88E-05
380	PLA2G10	ILMN_1762561	-0,22	-0,41	4,15E-04
381	PDCD10	ILMN_1709396	-0,22	-0,38	2,91E-04
382	CST6	ILMN_1697733	-0,21	-0,47	1,15E-04
383	FAM89A	ILMN_1652677	-0,21	-0,58	3,34E-04
384	DDR1	ILMN_1812262	-0,21	-0,67	2,20E-04
385	ITPR1	ILMN_1789505	-0,21	-0,45	1,38E-04
386	ST14	ILMN_1681245	-0,21	-0,44	4,67E-04
387	C14ORF151	ILMN_1729596	-0,21	-0,95	6,82E-05
388	ITGA3	ILMN_1685397	-0,20	-0,97	2,84E-05
389	NY-SAR-48	ILMN_1768020	-0,20	-0,52	3,70E-04
390	GPR114	ILMN_1666902	-0,20	-0,48	2,18E-04
391	ABHD12	ILMN_1745116	-0,20	-0,64	1,38E-04
392	ITGA2	ILMN_1665792	-0,19	-0,63	3,84E-04
393	HS.572030	ILMN_1879326	-0,18	-0,56	4,01E-04
394	GLRX	ILMN_1737308	-0,17	-0,65	1,15E-04
395	OAS1	ILMN_1658247	-0,17	-0,85	3,87E-04
396	CLDN9	ILMN_1740276	-0,17	-0,58	4,68E-04
397	CALU	ILMN_1727194	-0,17	-0,73	3,95E-05
398	SRPX2	ILMN_1676213	-0,17	-0,86	4,82E-05
399	ATP6V1A	ILMN_1711516	-0,16	-0,49	4,54E-04
400	GJB3	ILMN_1652390	-0,15	-0,77	4,00E-04
401	C12ORF36	ILMN_1694293	-0,13	-0,90	2,90E-05
402	GPX3	ILMN_1726666	-0,13	-0,74	9,58E-05
403	SPRR1A	ILMN_1711174	-0,13	-0,94	9,91E-06
404	CSF2	ILMN_1661861	-0,12	-0,71	1,15E-04
405	SDCBP2	ILMN_1749535	-0,10	-1,32	2,31E-06
406	DHRS9	ILMN_1727150	0,12	-1,79	4,10E-06

[1]Score=$(FC_{AC}+FC_{BC})/2$ – Stddev(FC_{AC},FC_{BC})

[2]LogFCmean=$(FC_{AC}+FC_{BC})/2$

FC=Fold Change, Stddev = Standard deviation
