# Supplementary material for: Expression profiling and intracellular localization studies of the novel Proline-, Histidine-, and Glycine-rich protein 1 suggest an essential role in gastro-intestinal epithelium and a potential clinical application in colorectal cancer diagnostics
Source: BMC Gastroenterol. 2018 Feb 7;18:26. doi: 10.1186/s12876-018-0752-8 (PMC5803922; doi:10.1186/s12876-018-0752-8)
Supplement: Supplementary file 6 — Downregulated genes. (PDF 79 kb) [file 12876_2018_752_MOESM6_ESM.pdf]

Additional file 6: Downregulated genes

No	ILMN_Gene	Probe_ID	Score[1]	logFCmean[2]	adjPmean
1	RASD1	ILMN_1740426	1,83	2,08	3,07E-10
2	SOSTDC1	ILMN_1715463	1,22	1,63	1,51E-09
3	DDIT4	ILMN_1661599	1,19	1,41	9,66E-10
4	LOC644844	ILMN_1661078	1,15	1,20	2,63E-08
5	ULBP1	ILMN_1738407	1,13	1,16	6,17E-08
6	ASS1	ILMN_1688234	1,12	1,29	1,32E-09
7	CLEC3A	ILMN_1761066	0,99	1,76	2,12E-09
8	SLC4A4	ILMN_1734897	0,99	1,15	3,37E-07
9	ALDH1L1	ILMN_1802167	0,91	1,04	1,86E-08
10	CTH	ILMN_1784112	0,91	0,97	1,82E-07
11	HS.389988	ILMN_1874575	0,90	1,03	3,56E-06
12	CHAC1	ILMN_1739241	0,88	0,88	6,97E-08
13	DEPDC6	ILMN_1756685	0,86	0,99	2,23E-07
14	F2R	ILMN_1742866	0,86	1,17	9,43E-08
15	ASNS	ILMN_1796417	0,85	0,91	9,40E-06
16	PTBP2	ILMN_1707240	0,85	1,31	1,40E-06
17	MAOA	ILMN_1663640	0,84	0,91	3,01E-06
18	PRSS1	ILMN_1734773	0,84	1,00	8,23E-08
19	PSPH	ILMN_1776105	0,81	0,91	1,02E-07
20	RGS2	ILMN_1710974	0,80	1,20	1,71E-06
21	PROM1	ILMN_1786720	0,77	1,01	5,77E-08
22	PCK2	ILMN_1760649	0,75	0,91	2,90E-08
23	HS.355933	ILMN_1915076	0,74	0,84	2,18E-07
24	MXD4	ILMN_1756541	0,72	0,72	1,27E-05
25	SCML1	ILMN_1776653	0,72	0,83	2,31E-05
26	CFTR	ILMN_1705813	0,71	1,14	1,07E-06
27	CAP2	ILMN_1691237	0,71	1,05	5,31E-07
28	RFX5	ILMN_1741200	0,70	0,87	7,78E-06
29	IVD	ILMN_1724207	0,69	0,72	1,27E-05
30	NFIX	ILMN_1694325	0,69	0,82	4,85E-05
31	GPC2	ILMN_1651642	0,68	0,91	3,80E-08
32	CCDC98	ILMN_1754344	0,68	0,87	1,91E-05
33	CEBPG	ILMN_1716766	0,68	0,68	5,69E-07
34	HOXB9	ILMN_1716708	0,67	0,76	5,95E-06
35	FLJ39822	ILMN_1709635	0,67	0,88	1,40E-06
36	HIST2H2BE	ILMN_1732071	0,67	1,33	5,03E-07
37	NUPR1	ILMN_1790234	0,66	0,71	1,47E-06
38	PTGS2	ILMN_1677511	0,66	1,12	1,59E-06
39	HIST1H2AG	ILMN_1686478	0,65	0,75	7,33E-07
40	TSGA14	ILMN_1797209	0,65	0,67	2,51E-05
41	IGSF10	ILMN_1673141	0,64	0,69	5,07E-07
42	ITPR2	ILMN_1736103	0,63	0,74	5,06E-06
43	TUBE1	ILMN_1764398	0,63	0,71	1,45E-05
44	ADM2	ILMN_1702933	0,63	0,83	8,18E-07
45	NMU	ILMN_1740250	0,63	0,66	1,29E-05
46	FZD4	ILMN_1743367	0,63	0,65	2,18E-06

Additional file 6: Downregulated genes

47	ALDH6A1	ILMN_1785284	0,62	0,87	3,08E-05
48	FILIP1L	ILMN_1738578	0,61	0,74	8,29E-07
49	BBS2	ILMN_1767612	0,61	0,68	2,10E-05
50	KIAA1199	ILMN_1813704	0,61	0,77	3,09E-06
51	CD99L2	ILMN_1700681	0,60	0,70	2,45E-06
52	EVI5L	ILMN_1747281	0,60	0,78	4,47E-07
53	KIAA0831	ILMN_1678808	0,60	0,68	1,70E-05
54	LOC727820	ILMN_1776327	0,59	0,65	4,51E-06
55	KIAA1468	ILMN_1798346	0,59	0,65	4,62E-05
56	ADH1C	ILMN_1740717	0,58	0,60	3,01E-06
57	HS.19339	ILMN_1856480	0,58	0,62	3,14E-06
58	ITGB6	ILMN_1662635	0,57	0,93	7,96E-05
59	MMP7	ILMN_1685403	0,57	0,92	5,80E-07
60	CEBPD	ILMN_1782050	0,57	0,60	2,41E-06
61	TSC22D3	ILMN_1748124	0,56	0,64	2,26E-05
62	CXCR4	ILMN_1683307	0,56	0,81	7,51E-06
63	DR1	ILMN_1759983	0,56	0,67	2,15E-06
64	EDN1	ILMN_1682775	0,56	0,60	6,67E-06
65	ADAMTS6	ILMN_1767362	0,55	1,01	5,72E-06
66	HIBADH	ILMN_1804150	0,55	0,57	1,28E-05
67	SPRYD3	ILMN_1735250	0,55	0,82	4,40E-05
68	C20ORF108	ILMN_1708016	0,55	0,69	1,00E-04
69	CARS	ILMN_1696066	0,55	0,69	7,33E-05
70	LOC641953	ILMN_1697232	0,55	0,83	3,21E-05
71	HOXC6	ILMN_1787334	0,55	0,61	1,64E-05
72	C14ORF135	ILMN_1680781	0,54	0,55	7,85E-06
73	KAZALD1	ILMN_1684755	0,54	0,67	1,85E-06
74	PPAPDC2	ILMN_1802628	0,54	0,60	5,15E-05
75	GARS	ILMN_1771026	0,53	0,56	1,69E-05
76	THNSL1	ILMN_1782688	0,53	0,65	1,40E-05
77	SLC25A13	ILMN_1668012	0,53	0,57	6,01E-05
78	SH3BGRL	ILMN_1702835	0,53	0,67	2,62E-06
79	CLUAP1	ILMN_1750596	0,53	0,58	2,37E-06
80	SCIN	ILMN_1813561	0,53	0,87	2,15E-06
81	WDR91	ILMN_1652223	0,53	0,54	8,37E-05
82	TEK	ILMN_1751576	0,52	0,57	8,01E-06
83	GNA14	ILMN_1734857	0,52	0,70	7,85E-07
84	MCCC1	ILMN_1760174	0,52	0,53	2,07E-05
85	AKAP7	ILMN_1679943	0,51	0,81	1,14E-06
86	PCCA	ILMN_1714384	0,51	0,54	9,27E-05
87	HS.526540	ILMN_1874735	0,50	0,51	9,20E-05
88	PAM	ILMN_1788631	0,50	0,63	2,74E-06
89	LOC643888	ILMN_1665060	0,50	0,51	2,12E-05
90	APOLD1	ILMN_1723522	0,50	0,70	9,71E-07
91	TMEM117	ILMN_1809894	0,50	0,57	2,83E-05
92	SNX10	ILMN_1786257	0,50	0,56	9,29E-06
93	HS.550806	ILMN_1869189	0,50	0,63	4,60E-04

Additional file 6: Downregulated genes

94	CBX7	ILMN_1657361	0,50	0,58	4,95E-06
95	MGC24039	ILMN_1791593	0,50	0,59	2,28E-05
96	WDR45L	ILMN_1719140	0,50	0,50	1,54E-04
97	C11ORF71	ILMN_1746091	0,50	0,58	6,07E-06
98	FLJ12355	ILMN_1697115	0,50	0,50	2,09E-05
99	GNAI1	ILMN_1742044	0,49	0,82	5,33E-06
100	C5ORF13	ILMN_1680738	0,49	0,52	5,87E-05
101	PCCB	ILMN_1761010	0,49	0,54	5,71E-06
102	PSAT1	ILMN_1692938	0,49	0,55	7,86E-06
103	DLD	ILMN_1664577	0,49	0,62	4,54E-05
104	ANKMY2	ILMN_1784292	0,48	0,64	9,50E-05
105	NDRG3	ILMN_1738229	0,48	0,57	2,14E-04
106	MTHFD1L	ILMN_1772521	0,48	0,53	1,06E-05
107	RHOQ	ILMN_1810559	0,48	0,56	2,28E-05
108	CCDC71	ILMN_1768433	0,48	0,48	4,76E-05
109	FLJ40142	ILMN_1753265	0,48	0,75	1,61E-06
110	ZCCHC7	ILMN_1744980	0,48	0,48	1,03E-05
111	C9ORF91	ILMN_1705313	0,47	0,48	2,85E-05
112	SLC37A3	ILMN_1671704	0,47	0,61	1,07E-05
113	KLF15	ILMN_1683133	0,47	0,55	9,74E-06
114	LOC153364	ILMN_1769449	0,47	0,47	4,90E-05
115	LOC652458	ILMN_1718353	0,47	0,54	2,19E-04
116	AARS	ILMN_1662364	0,47	0,53	4,03E-05
117	COPG2	ILMN_1722065	0,47	0,63	1,21E-05
118	GBAS	ILMN_1778611	0,47	0,59	6,70E-05
119	C14ORF159	ILMN_1721106	0,47	0,47	5,24E-05
120	PIR	ILMN_1761247	0,46	0,55	4,87E-05
121	ONECUT2	ILMN_1664462	0,46	0,57	4,97E-05
122	XYLB	ILMN_1794349	0,46	0,56	3,43E-04
123	SP4	ILMN_1721081	0,46	0,48	1,77E-04
124	STK38L	ILMN_1755792	0,45	0,56	3,53E-05
125	ZNF32	ILMN_1673112	0,45	0,51	9,01E-06
126	BCL6	ILMN_1737314	0,45	0,46	5,52E-05
127	INHBE	ILMN_1811767	0,45	0,52	8,62E-06
128	TGIF2	ILMN_1709044	0,45	0,52	1,01E-05
129	VASH2	ILMN_1692698	0,45	0,62	8,68E-06
130	HS.505676	ILMN_1821280	0,45	0,60	6,77E-06
131	DIXDC1	ILMN_1736704	0,45	0,53	4,85E-06
132	MLF1	ILMN_1684439	0,44	0,45	4,76E-04
133	POLR3GL	ILMN_1760667	0,44	0,53	3,50E-05
134	LOC400657	ILMN_1725528	0,44	0,52	4,83E-05
135	MSH3	ILMN_1719471	0,44	0,45	4,24E-04
136	VASN	ILMN_1667295	0,44	0,61	5,17E-06
137	LOC644311	ILMN_1652259	0,44	0,46	3,55E-05
138	MT1G	ILMN_1715401	0,44	0,48	3,86E-04
139	ATG10	ILMN_1748968	0,44	0,58	1,33E-05
140	RHBDL2	ILMN_1685330	0,44	0,46	2,27E-04

Additional file 6: Downregulated genes

141	RWDD2A	ILMN_1722156	0,44	0,57	5,12E-05
142	PIGW	ILMN_1743397	0,43	0,48	4,86E-04
143	EVI1	ILMN_1803367	0,43	0,44	4,28E-05
144	LOC644869	ILMN_1665571	0,43	0,48	8,00E-05
145	TCEAL3	ILMN_1749478	0,43	0,51	1,63E-05
146	LMO4	ILMN_1703487	0,43	0,58	2,26E-06
147	HTATSF1	ILMN_1683221	0,43	0,51	5,76E-06
148	USP13	ILMN_1708059	0,43	0,45	1,53E-04
149	ADRB2	ILMN_1695590	0,43	0,47	1,54E-05
150	PQLC3	ILMN_1814213	0,43	0,49	2,21E-04
151	PHGDH	ILMN_1704537	0,43	0,75	3,69E-06
152	INTS3	ILMN_1756086	0,42	0,48	1,08E-04
153	FAM113A	ILMN_1676302	0,42	0,47	2,46E-05
154	ATF3	ILMN_1661109	0,42	0,53	1,89E-04
155	HS.7572	ILMN_1863185	0,42	0,53	3,61E-05
156	HS.107801	ILMN_1863009	0,42	0,53	1,96E-05
157	C6ORF48	ILMN_1766446	0,42	0,53	2,13E-05
158	CORO1C	ILMN_1745954	0,42	0,50	2,15E-04
159	C6ORF117	ILMN_1794829	0,42	0,81	3,42E-06
160	VIPR1	ILMN_1707959	0,41	0,49	2,93E-05
161	IMMP2L	ILMN_1809292	0,41	0,41	1,42E-04
162	ACOT4	ILMN_1764321	0,41	0,47	3,57E-05
163	CEP110	ILMN_1758778	0,41	0,52	4,33E-05
164	DNAJA4	ILMN_1776998	0,41	0,53	3,94E-05
165	ANKRD50	ILMN_1729342	0,41	0,52	1,21E-04
166	YARS	ILMN_1766010	0,41	0,47	6,64E-05
167	FLJ22662	ILMN_1736605	0,41	0,59	1,14E-05
168	PTPN13	ILMN_1652805	0,41	0,48	1,66E-04
169	CIT	ILMN_1680649	0,41	0,43	1,08E-04
170	ZNF643	ILMN_1799086	0,41	0,48	4,27E-04
171	DYNC1I1	ILMN_1690397	0,41	0,56	8,45E-05
172	TBC1D9	ILMN_1703891	0,41	0,76	2,58E-06
173	TIGD2	ILMN_1690066	0,40	0,44	4,14E-04
174	USP52	ILMN_1658373	0,40	0,58	4,88E-05
175	P2RY5	ILMN_1786429	0,40	0,49	7,03E-06
176	GTF2E1	ILMN_1655921	0,40	0,44	8,76E-05
177	PHF20L1	ILMN_1727287	0,40	0,68	2,48E-05
178	TNFSF10	ILMN_1801307	0,40	0,55	1,59E-05
179	KIAA0652	ILMN_1692306	0,40	0,41	3,71E-04
180	HS.405877	ILMN_1875354	0,39	0,43	1,28E-04
181	NHS	ILMN_1715864	0,39	0,51	1,72E-05
182	SCARNA9	ILMN_1805064	0,39	0,65	1,01E-05
183	DLG7	ILMN_1749829	0,39	0,46	1,79E-04
184	DKFZP564J102	ILMN_1786278	0,39	0,46	1,01E-04
185	ZFAND6	ILMN_1778803	0,39	0,41	3,35E-04
186	C20ORF127	ILMN_1730049	0,39	0,42	3,53E-05
187	STMN1	ILMN_1657796	0,39	0,55	1,85E-04

Additional file 6: Downregulated genes

188	DOCK1	ILMN_1715789	0,39	0,44	3,09E-04
189	FBXL17	ILMN_1715901	0,39	0,44	3,31E-05
190	ETV5	ILMN_1669756	0,39	0,48	1,01E-05
191	DDC	ILMN_1811601	0,39	0,50	2,16E-04
192	MOSC2	ILMN_1665775	0,39	0,58	1,64E-04
193	C9ORF97	ILMN_1684520	0,39	0,47	5,19E-05
194	ALDH7A1	ILMN_1763461	0,38	0,47	6,31E-06
195	KBTBD7	ILMN_1731188	0,38	0,49	8,70E-05
196	XPC	ILMN_1790807	0,38	0,48	1,24E-04
197	CELSR3	ILMN_1691290	0,38	0,54	6,97E-05
198	BIVM	ILMN_1746666	0,38	0,49	1,50E-04
199	DYNC2H1	ILMN_1809954	0,38	0,47	6,59E-05
200	C5ORF21	ILMN_1654542	0,38	0,41	4,18E-04
201	LOC645733	ILMN_1672656	0,38	0,44	2,39E-05
202	CCDC121	ILMN_1666995	0,38	0,40	1,69E-04
203	ITFG2	ILMN_1701244	0,37	0,40	2,20E-04
204	CABP4	ILMN_1688864	0,37	0,40	8,11E-05
205	C1ORF66	ILMN_1791762	0,37	0,40	1,27E-04
206	BTN3A2	ILMN_1700067	0,37	0,52	7,71E-05
207	BRMS1L	ILMN_1775943	0,37	0,47	2,21E-04
208	ST3GAL5	ILMN_1713496	0,37	0,38	4,30E-04
209	KDELC2	ILMN_1651557	0,37	0,43	1,17E-05
210	JAG2	ILMN_1764729	0,37	0,39	1,10E-04
211	ZNF532	ILMN_1731358	0,37	0,47	5,58E-05
212	TDRD3	ILMN_1745811	0,37	0,44	2,88E-04
213	TIGA1	ILMN_1654609	0,37	0,58	3,80E-05
214	CEP70	ILMN_1741350	0,37	0,44	1,97E-04
215	ZNF616	ILMN_1719219	0,37	0,65	5,56E-05
216	NPAT	ILMN_1772165	0,36	0,44	3,05E-04
217	HOXC4	ILMN_1719975	0,36	0,47	3,95E-05
218	LOC653066	ILMN_1759870	0,36	0,40	2,20E-04
219	LANCL1	ILMN_1703697	0,36	0,40	4,87E-04
220	TRIB1	ILMN_1767754	0,36	0,42	5,89E-05
221	FSCN1	ILMN_1808707	0,36	0,44	3,41E-04
222	HES1	ILMN_1710284	0,36	0,49	1,06E-04
223	TCEAL4	ILMN_1748625	0,36	0,48	2,96E-05
224	JUND	ILMN_1810214	0,36	0,63	1,28E-05
225	TTC30A	ILMN_1735822	0,35	0,39	1,32E-04
226	RETSAT	ILMN_1789961	0,35	0,37	1,66E-04
227	LARS	ILMN_1757317	0,35	0,45	3,68E-05
228	CTNNBIP1	ILMN_1688103	0,35	0,48	1,58E-04
229	HDAC6	ILMN_1798546	0,35	0,46	5,91E-05
230	MTMR4	ILMN_1771019	0,35	0,43	4,74E-04
231	AGK	ILMN_1772645	0,35	0,42	1,32E-04
232	GYS1	ILMN_1711289	0,35	0,39	1,67E-04
233	PPP1R9A	ILMN_1785680	0,35	0,51	2,34E-04
234	DAP3	ILMN_1781680	0,35	0,35	4,29E-04

Additional file 6: Downregulated genes

235	HS.10862	ILMN_1843198	0,35	0,50	3,57E-04
236	MIPEP	ILMN_1669070	0,35	0,50	4,48E-05
237	ETS2	ILMN_1745578	0,35	0,49	3,69E-05
238	PRR11	ILMN_1782403	0,35	0,45	2,21E-04
239	MRP63	ILMN_1774312	0,35	0,43	6,01E-05
240	ABLIM1	ILMN_1785424	0,34	0,35	2,65E-04
241	FRAP1	ILMN_1769031	0,34	0,35	2,69E-04
242	MGC70857	ILMN_1693862	0,34	0,36	5,28E-04
243	DHTKD1	ILMN_1664369	0,34	0,42	4,04E-04
244	HS.560167	ILMN_1881424	0,34	0,43	1,00E-04
245	SPG11	ILMN_1665049	0,34	0,50	1,86E-04
246	FOXA2	ILMN_1763712	0,34	0,37	2,78E-04
247	LOC730256	ILMN_1704014	0,34	0,60	1,61E-05
248	C19ORF46	ILMN_1801697	0,34	0,55	1,54E-05
249	KIF15	ILMN_1809440	0,33	0,37	4,48E-04
250	HS.556255	ILMN_1886515	0,33	0,37	2,31E-04
251	DARS	ILMN_1813836	0,33	0,35	3,15E-04
252	C18ORF56	ILMN_1796074	0,33	0,43	3,36E-04
253	COCH	ILMN_1711514	0,33	0,41	1,03E-04
254	ALDH16A1	ILMN_1776384	0,33	0,43	2,01E-04
255	GTDC1	ILMN_1762741	0,33	0,38	1,26E-04
256	OMA1	ILMN_1670079	0,33	0,34	1,32E-04
257	ZNF295	ILMN_1782110	0,33	0,52	8,02E-05
258	MYB	ILMN_1711894	0,33	0,40	8,89E-05
259	ASPM	ILMN_1815184	0,33	0,43	2,05E-04
260	LOC440925	ILMN_1663081	0,33	0,52	2,12E-05
261	BICD2	ILMN_1651375	0,33	0,36	3,15E-04
262	MGC16169	ILMN_1769601	0,33	0,47	1,88E-04
263	KIF2A	ILMN_1734476	0,32	0,35	2,94E-04
264	NUBPL	ILMN_1750412	0,32	0,37	2,57E-04
265	SMARCA4	ILMN_1814173	0,32	0,37	1,88E-04
266	TGIF1	ILMN_1702211	0,32	0,35	1,43E-04
267	BAMBI	ILMN_1691410	0,32	0,45	2,69E-04
268	SFXN2	ILMN_1795976	0,32	0,41	7,26E-05
269	SEMA4F	ILMN_1805007	0,32	0,40	2,01E-04
270	HS.436379	ILMN_1857897	0,32	0,36	3,98E-04
271	HS.4988	ILMN_1816244	0,32	0,58	8,03E-05
272	NEIL2	ILMN_1715680	0,32	0,36	3,57E-04
273	ASH2L	ILMN_1666933	0,32	0,39	6,35E-05
274	PPP1R3F	ILMN_1784822	0,32	0,42	2,67E-04
275	NFIB	ILMN_1778991	0,31	0,49	1,02E-04
276	DPY19L3	ILMN_1725072	0,31	0,53	3,12E-04
277	WDR27	ILMN_1688160	0,31	0,52	8,65E-05
278	SHMT2	ILMN_1661264	0,31	0,43	6,99E-05
279	NGRN	ILMN_1660345	0,31	0,37	3,41E-04
280	C15ORF15	ILMN_1803489	0,31	0,35	5,45E-04
281	TTC23	ILMN_1690320	0,31	0,66	1,15E-04

Additional file 6: Downregulated genes

282	TLOC1	ILMN_1762003	0,31	0,50	9,38E-05
283	ATP6V1B1	ILMN_1812073	0,31	0,40	4,93E-04
284	SLC7A1	ILMN_1683859	0,31	0,34	5,88E-04
285	LTA4H	ILMN_1690342	0,30	0,39	9,61E-05
286	ZKSCAN1	ILMN_1727603	0,30	0,54	2,34E-04
287	PDCD4	ILMN_1768004	0,30	0,35	4,55E-04
288	ATRN	ILMN_1678541	0,30	0,39	2,38E-04
289	LMCD1	ILMN_1754969	0,30	0,34	4,24E-04
290	VSIG2	ILMN_1666536	0,30	0,39	2,12E-04
291	FBXO4	ILMN_1755811	0,30	0,31	4,86E-04
292	MTIF2	ILMN_1791403	0,30	0,37	4,73E-04
293	LOC400566	ILMN_1707137	0,30	0,34	8,83E-05
294	OPLAH	ILMN_1711030	0,30	0,33	2,37E-04
295	SRD5A1	ILMN_1793241	0,30	0,53	7,70E-05
296	ACOT8	ILMN_1679600	0,30	0,33	1,60E-04
297	C14ORF105	ILMN_1807667	0,30	0,49	4,32E-04
298	ZNF318	ILMN_1792305	0,30	0,39	1,18E-04
299	HS.372654	ILMN_1829989	0,30	0,44	4,86E-04
300	HS.92308	ILMN_1817255	0,30	0,34	4,58E-04
301	BTN3A1	ILMN_1802708	0,30	0,33	3,60E-04
302	LOC153684	ILMN_1782753	0,30	0,36	2,49E-04
303	ATP9B	ILMN_1658684	0,30	0,39	3,44E-04
304	PLCL2	ILMN_1737025	0,30	0,64	5,64E-05
305	CFD	ILMN_1777190	0,30	0,37	3,69E-04
306	DDEFL1	ILMN_1685441	0,30	0,59	9,29E-06
307	SETDB2	ILMN_1731644	0,29	0,36	4,63E-04
308	KIAA1600	ILMN_1752927	0,29	0,40	3,14E-04
309	WDR71	ILMN_1652753	0,29	0,32	2,01E-04
310	EIF3S7	ILMN_1739847	0,29	0,30	6,24E-04
311	HS.567841	ILMN_1895276	0,29	0,46	6,94E-05
312	PIGZ	ILMN_1790352	0,29	0,61	6,37E-05
313	SDHA	ILMN_1744210	0,29	0,32	3,37E-04
314	API5	ILMN_1815051	0,29	0,32	5,89E-04
315	TANC1	ILMN_1772278	0,29	0,59	2,25E-04
316	OPN3	ILMN_1716988	0,29	0,47	2,61E-04
317	LOC730746	ILMN_1679450	0,29	0,45	5,78E-04
318	STAT5B	ILMN_1777783	0,29	0,31	4,35E-04
319	HIST1H2BD	ILMN_1651496	0,29	0,53	5,45E-05
320	MAGED2	ILMN_1683576	0,29	0,29	5,48E-04
321	FLJ20444	ILMN_1811181	0,28	0,31	3,63E-04
322	MGC52110	ILMN_1683065	0,28	0,38	4,21E-04
323	CCDC109B	ILMN_1801766	0,28	0,34	4,53E-04
324	HOXA6	ILMN_1815570	0,28	0,40	3,85E-04
325	MPHOSPH9	ILMN_1654421	0,28	0,47	3,60E-04
326	ACAT1	ILMN_1800008	0,28	0,36	5,15E-04
327	ERN1	ILMN_1698404	0,28	0,32	4,39E-04
328	NLN	ILMN_1742089	0,28	0,34	2,92E-04

Additional file 6: Downregulated genes

329	NARS2	ILMN_1797332	0,28	0,45	1,99E-04
330	GOLSYN	ILMN_1738989	0,28	0,33	4,10E-04
331	ALDH2	ILMN_1793859	0,27	0,31	3,18E-04
332	RNF41	ILMN_1700345	0,27	0,47	3,01E-04
333	HIST1H3G	ILMN_1726815	0,27	0,35	2,63E-04
334	LOC650215	ILMN_1743711	0,27	0,34	2,75E-04
335	CCT6B	ILMN_1699610	0,27	0,31	7,76E-04
336	LOC647340	ILMN_1685774	0,27	0,46	1,55E-04
337	ZBED3	ILMN_1683215	0,27	0,38	3,25E-04
338	SLC43A1	ILMN_1765118	0,27	0,43	2,27E-04
339	MAGED1	ILMN_1762792	0,27	0,52	4,17E-04
340	ALDH3A1	ILMN_1702503	0,27	0,76	3,37E-04
341	AASDH	ILMN_1784269	0,26	0,31	3,22E-04
342	WDR6	ILMN_1669484	0,26	0,30	8,75E-04
343	DYM	ILMN_1680130	0,26	0,34	2,59E-04
344	LOC283932	ILMN_1710954	0,26	0,54	1,10E-04
345	NMT2	ILMN_1656378	0,25	0,35	3,35E-04
346	RRM2B	ILMN_1747763	0,25	0,74	2,19E-04
347	HS.571151	ILMN_1914800	0,24	0,61	3,04E-04
348	MAPK12	ILMN_1797786	0,24	0,40	1,54E-04
349	PRKCA	ILMN_1771800	0,24	0,46	4,23E-04
350	ZYG11B	ILMN_1656676	0,24	0,28	5,38E-04
351	CENPE	ILMN_1716279	0,24	0,55	4,77E-04
352	PPP3CB	ILMN_1802669	0,23	0,61	3,32E-04
353	EPB41L5	ILMN_1770245	0,23	0,29	4,45E-04
354	HIST1H2AC	ILMN_1792689	0,23	0,79	1,02E-04
355	LCN2	ILMN_1692223	0,23	0,35	4,78E-04
356	KLHL9	ILMN_1664466	0,23	0,41	3,80E-04
357	PHYH	ILMN_1773073	0,23	0,40	1,91E-04
358	HS.445274	ILMN_1911717	0,23	0,54	3,88E-04
359	CCNB2	ILMN_1801939	0,23	0,35	2,81E-04
360	HAGHL	ILMN_1793201	0,22	0,35	3,11E-04
361	CYP1B1	ILMN_1693338	0,22	0,72	1,13E-04
362	ZNF334	ILMN_1672743	0,22	0,46	4,96E-04
363	SH2D2A	ILMN_1766319	0,22	0,54	5,83E-05
364	BBS1	ILMN_1800590	0,21	0,64	4,37E-04
365	ZNF294	ILMN_1760360	0,21	0,70	4,59E-04
366	SIX5	ILMN_1793672	0,20	0,47	3,32E-04
367	HEBP1	ILMN_1802557	0,20	0,39	2,33E-04
368	CABC1	ILMN_1731064	0,19	0,45	5,03E-04
369	SH2B3	ILMN_1752046	0,14	0,62	3,14E-04

[1]Score=$(FC_{AC}+FC_{BC})/2$ – Stddev(FC_{AC},FC_{BC})

[2]LogFCmean=$(FC_{AC}+FC_{BC})/2$

FC=Fold Change, Stddev = Standard deviation
