# Supplementary material for: Expression profiling and intracellular localization studies of the novel Proline-, Histidine-, and Glycine-rich protein 1 suggest an essential role in gastro-intestinal epithelium and a potential clinical application in colorectal cancer diagnostics
Source: BMC Gastroenterol. 2018 Feb 7;18:26. doi: 10.1186/s12876-018-0752-8 (PMC5803922; doi:10.1186/s12876-018-0752-8)
Supplement: Supplementary file 7 — Gene ontology analysis. (PDF 41 kb) [file 12876_2018_752_MOESM7_ESM.pdf]

Additional file 7: Gene ontology analysis

GO category	Up/Down	Overrepresented functional annotation	GO ID	Genes in GO	Genes in list	Percent	P-value	Adj. P-value
GO_BP	Up	Establishment of localization	GO:0051234	95	356	26.7	2.5E-7	1.7E-4
GO_BP	Up	Transport	GO:0006810	94	356	26.4	2.4E-7	2.4E-4
GO_BP	Up	Localization	GO:0051179	102	356	28.7	2.1E-7	4.2E-4
GO_BP	Down	Cellular amino acid metabolic process	GO:0006520	19	303	6.3	1.1E-6	5.7E-4
GO_BP	Down	Organic acid metabolic process	GO:0006082	32	303	10.6	6.5E-7	6.7E-4
GO_BP	Down	Cellular ketone metabolic process	GO:0042180	32	303	10.6	1.0E-6	7.0E-4
GO_BP	Down	Carboxylic acid metabolic process	GO:0019752	32	303	10.6	5.0E-7	1.0E-3
GO_BP	Down	Oxoacid metabolic process	GO:0043436	32	303	10.6	5.0E-7	1.0E-3
GO_BP	Down	Cellular amine metabolic process	GO:0044106	21	303	6.9	2.8E-6	1.1E-3
GO_BP	Down	System development	GO:0048731	62	303	20.5	1.3E-5	4.5E-3
GO_BP	Down	Cellular amino acid and derivatives met. proc.	GO:0006519	21	303	6.9	3.4E-5	1.0E-2
GO_BP	Down	Anatomical structure development	GO:0048856	65	303	21.5	4.8E-5	1.3E-2
GO_BP	Down	Amine metabolic process	GO:0009308	21	303	6.9	6.6E-5	1.5E-2
GO_BP	Down	Developmental process	GO:0032502	76	303	25.1	7.4E-5	1.5E-2
GO_BP	Down	G-protein coupled rec. pr. signaling pathway	GO:0007186	16	303	5.3	1.0E-4	1.9E-2
GO_BP	Down	Cell differentiation	GO:0030154	44	303	14.5	1.5E-4	2.5E-2
GO_BP	Down	Cellular developmental process	GO:0048869	46	303	15.2	1.7E-4	2.6E-2
GO_BP	Down	Multicellular organismal process	GO:0032501	82	303	27.1	1.9E-4	2.9E-2
GO_BP	Down	Multicellular organismal devel.	GO:0007275	67	303	22.1	2.6E-4	3.5E-2
GO_CC	Up	Plasma membrane	GO:0005886	107	356	30.1	3.7E-12	6.5E-10
GO_CC	Up	Membrane part	GO:0044425	178	356	50.0	3.7E-12	1.3E-9
GO_CC	Up	Membrane	GO:0016020	193	356	54.2	3.5E-11	4.0E-9
GO_CC	Up	Plasma membrane part	GO:0044459	72	356	20.2	1.1E-8	9.3E-7
GO_CC	Up	Extracellular region	GO:0005576	49	356	13.8	2.4E-8	1.4E-6
GO_CC	Up	Intrinsic to membrane	GO:0031224	135	356	37.9	2.1E-8	1.5E-6
GO_CC	Up	Integral to membrane	GO:0016021	131	356	36.8	4.0E-8	2.0E-6
GO_CC	Up	Cell surface	GO:0009986	17	356	4.8	7.9E-5	3.4E-3
GO_CC	Up	Cell fraction	GO:0000267	42	356	11.8	5.0E-4	1.9E-2
GO_CC	Up	Intrinsic to plasma membrane	GO:0031226	31	356	8.7	8.0E-4	2.8E-2
GO_CC	Up	Endoplasmic reticulum part	GO:0044432	22	356	6.2	1.0E-3	3.0E-2
GO_CC	Up	Endoplasmic reticulum	GO:0005783	44	356	12.4	9.6E-4	3.0E-2
GO_CC	Up	Integral to plasma membrane	GO:0005887	30	356	8.4	1.2E-3	3.1E-2
GO_CC	Up	Proton-transporting V-type ATPase complex	GO:0033176	5	356	1.4	1.6E-3	3.9E-2
GO_CC	Up	Insoluble fraction	GO:0005626	32	356	9.0	2.3E-3	4.9E-2
