# Supplementary material for: Expression profiling and intracellular localization studies of the novel Proline-, Histidine-, and Glycine-rich protein 1 suggest an essential role in gastro-intestinal epithelium and a potential clinical application in colorectal cancer diagnostics
Source: BMC Gastroenterol. 2018 Feb 7;18:26. doi: 10.1186/s12876-018-0752-8 (PMC5803922; doi:10.1186/s12876-018-0752-8)
Supplement: Supplementary file 8 — Clinicopathological data for the colorectal cancer patient cohort. (PDF 26 kb) [file 12876_2018_752_MOESM8_ESM.pdf]

Patient characteristics

Parameter	Value
**Median age [years] (range)**	76 (21-93)
**Gender – no. (%)**	
Female	117 (56)
Male	92 (44)
**Tumor stage – no. (%)**	
pT1	12 (6)
pT2	36 (17)
pT3	150 (72)
pT4	11 (5)
**Lymph node metastasis – no. (%)**	
pN0	145 (69)
pN1	48 (23)
pN2	15 (7)
Unknown	1 (0)
**Distant metastasis – no. (%)**	
M0	209 (100)
M1	0 (0)
**Tumor differentiation grade – no. (%)**	
I	11 (5)
II	146 (70)
III	52 (25)

Aditional file 8: Clinicopathological data for the colorectal cancer patient cohort
