# Supplementary material for: Expression profiling and intracellular localization studies of the novel Proline-, Histidine-, and Glycine-rich protein 1 suggest an essential role in gastro-intestinal epithelium and a potential clinical application in colorectal cancer diagnostics
Source: BMC Gastroenterol. 2018 Feb 7;18:26. doi: 10.1186/s12876-018-0752-8 (PMC5803922; doi:10.1186/s12876-018-0752-8)
Supplement: Supplementary file 1 — PHGR1 peptide sequence analysis. (PDF 31 kb) [file 12876_2018_752_MOESM1_ESM.pdf]

A

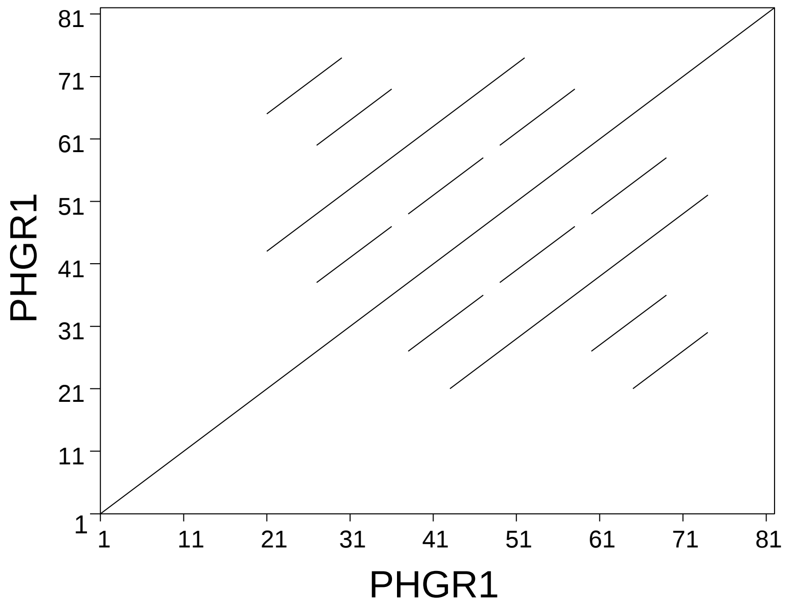

B

```
1         10        20        30        40
MDPGPKGHCHCGGHGHPPGHCGPPPGHGPGPCGPPPHHGPGP

        50        60        70        80
CGPPPGHGPGPCGPPPHHGPGPCGPPPGHGPGHPPPGPHH
```

Additional file 1: PHGR1 peptide sequence analysis.

A) Dotplot analysis of the human PHGR1 peptide sequence against itself. The window size was 10 residues.

B) Human PHGR1 peptide sequence. The five repeats are shown by colored boxes and underlining. The two variants of the repeated unit are indicated by different color and underlining.
